# The abundance and diversity of antibiotic resistance genes in layer chicken ceca is associated with farm enviroment

**DOI:** 10.3389/fmicb.2023.1177404

**Published:** 2023-06-30

**Authors:** Shasha Xiao, Jiandui Mi, Yingxin Chen, Kunxian Feng, Liang Mei, Xindi Liao, Yinbao Wu, Yan Wang

**Affiliations:** ^1^College of Animal Science, South China Agricultural University, Guangzhou, China; ^2^Guangdong Provincial Key Lab of Agro-Animal Genomics and Molecular Breeding, South China Agricultural University, Guangzhou, China; ^3^National Engineering Research Center for Breeding Swine Industry, College of Animal Science, South China Agricultural University, Guangzhou, China; ^4^Heyuan Branch, Guangdong Laboratory for Lingnan Modern Agriculture, Heyuan, China

**Keywords:** industrialized feedlot, lohmann layer chicken, cecal microbiome, antibiotic resistance genes, source tracker

## Abstract

Industrialized layer chicken feedlots harbor complex environmental microbial communities that affect the enrichment and exchange of gut bacteria and antibiotic resistance genes (ARGs). However, the contribution of different environmental sources to the gut ARGs of layer chickens is not clear. Here, layer chicken gut and environmental samples (air, water, feed, cage, feather, maternal hen feces, uropygial glands) were collected during the early 3 month period before the laying of eggs, and the source and characteristics of the gut microorganisms and ARGs were analyzed by performing 16S rRNA and metagenomic sequencing. The results showed that the abundances of Bacteroidetes and Actinobacteria in cecum of layer chickens gradually increased, while that of Proteobacteria decreased with age, and the number and relative abundance of ARGs decreased significantly with age. On day 5, 57% of the layer chicken cecal ARGs were from feather samples, and 30% were from cage samples. Subsequently, the contribution of cage ARGs became progressively more prominent over time. At days 30 and 57, the contribution of cage ARGs to the chick cecal ARGs reached 63.3 and 69.5%, respectively. The bacterial community composition (especially the abundances of *Klebsiella pneumoniae* and *Escherichia coli*) was the major factor impacting the ARG profile. *K. pneumoniae* and *E. coli* were mainly transmitted from feathers to the layer chicken cecum, and the contribution rates were 32 and 3.4%, respectively. In addition, we observed the transmission of ARG-carrying bacteria (*Bacteroides fragilis*) from the cage to the gut, with a contribution rate of 11.5%. It is noteworthy that *B. fragilis* is an opportunistic pathogen that may cause diarrhea in laying hens. These results can provide reference data for the healthy breeding of layer chickens and the prevention and control of ARG pollution.

## 1. Introduction

Antibiotic resistance genes (ARGs) are emerging environmental contaminants that have raised serious public health concerns. Approximately 700,000 people die every year because of resistant infections, and by 2050, that number will increase to 10 million deaths a year (Chin et al., 2018). It has been reported that approximately 73% of antibiotics use occurs in the farming industry (Van Boeckel et al., 2019). Antibiotics are administered to animals, usually through feed or drinking water for the prevention and treatment of diseases (Hu and Cheng, 2016). Therefore, the gut microbes of food animals likely constitute a large reservoir for ARGs, significantly increasing the risk of ARGs transmission through the food chain (Wang et al., 2019). However little attention has been paid to the gut microbial composition and associated ARGs in laying chicken, in contrast to the extensive studies of pig and broiler microbiomes and ARGs (He et al., 2014; Zhao et al., 2018). Consequently, a comprehensive study on the structure and function of the bacterial community composition and its associated ARGs in laying chickens is urgently needed.

The ARGs in the gut microbiota are mainly affected by bacterial community composition (Li et al., 2021). As the primary host of ARGs, bacterial communities account for 4.8–32.2% of the variation in the ARG profile (Pu et al., 2020; Yang et al., 2021). The establishment of the laying chicken gut microbiota is a complex and dynamic process (Diaz Carrasco et al., 2019). Age is an important factor affecting the composition of the gut microbiota. Early life is a critical window period for the colonization of gut microbiota in animals (Liu et al., 2019). The gut microbiota that is established during this early stage has an important influence on the growth of the animals and the development of the immune system (Tamburini et al., 2016). Therefore, it is helpful to further understand the association between changes in the gut microbiota and ARGs in laying chicken by studying the changes in gut microbiota over time in early life.

Despite evidence suggesting that newborn laying chicks acquire their initial microbial community from their maternal hen and immediate environment, the impact and relative contribution of different microbial sources in shaping the gut microbiota in layer chickens remains poorly understood (Lee et al., 2019; Maki et al., 2020). Research has shown that *Halomonas* and *Ochrobactrum* are dominant genera in embryos, and there was a moderate correlation (0.4) between the maternal hen and the embryo (Ding et al., 2017). The results suggest that the maternal hen fecal microbiota on eggshells may contribute to the establishment of gut microbiota in chicks. In addition, the rearing environment also exerts a sustained influence on the development of infant gut microbiota. The microbiota of indoor hens consists of a higher number of bacterial species than the microbiota of outdoor hens (Seidlerova et al., 2020). However, no longitudinal studies have been conducted to analyze the relative contributions of these bacterial sources to the colonization of newborn laying chicks.

Thus, a large-scale study was conducted to investigate the spatial and temporal development of cecal bacteria and ARGs in healthy newborn laying chicks, and to explore the relationship between bacterial transmission and the changes in ARGs within the gut microbiota. In addition, relative contributions of different microbial sources from the maternal hen fecal and the rearing environment (air, water, chicken cages, chicken feed, uropygial glands, and feathers) were also assessed.

## 2. Materials and methods

### 2.1. Sample collection

The layer chicken management was performed according to our previous study, we chose to sample more frequently during the early stages of rapid development and changes in the gut microbiota (0–7 days), and then transition to less frequent sampling during the later stages of community stabilization (Xiao et al., 2021). Random samples (5 g) of feed (day 0, 12, 43) were taken from the feed trough using sterile spoons and placed into sterile sampling bags. Six layer chickens were randomly obtained from the flock in the middle of house at days 0, 1, 3, 5, 7, 12, 18, 24, 30, 36, 40, 43, 50, and 57 after hatching, with a total of 84 layer chickens used for this study (Supplementary Figure S1). Using sterile scissors, 8–10 feathers were cut from the neck, back, abdomen, wings, and tail of the chickens, and mixed together in a sterile bag. And than the layer chicken selected for each time point were killed by cervical dislocation. After the abdomen was opened; the caecum was removed from each chicken, and immediately placed in liquid nitrogen and collected, transferred to the laboratory and stored at −80°C until DNA extraction. At the same time, maternal fecal samples were collected rectally from each laying hen using a sterile cotton swab (Hua Chen Yang, Shen Zhen, China) premoistened with sterile phosphate-buffered saline (PBS), and then the swab head was placed in a 5 mL sterile screw top collection tube (Corning, NY, United States). The uropygial gland was cut off with sterilized scissors and placed in a 5 mL sterile screw-top collection tube. Three water samples (approximately 3 L each) were collected from the water trough and placed into sterile containers at each sampling timepoint. Indoor air samples were collected using liquid-based air samplers, which were placed ~50 cm above the floor. Three replicate air samples were collected at 8:00, 12:00 and 16:00 by drawing air for 1 h at a rate of 13 L per min through impingers filled with sterile molecular-grade water. For each replicate, the air sample was collected three times simultaneously at five points indoors, and the resulting samples (30 mL each) were pooled (150 mL total volume). Cage surface samples from each coop were collected from six sites. During this procedure, a swab premoistened with sterile PBS was rubbed back and forth several times at each sampling site, and then the swab head was placed in a 5-mL sterile screw-top collection tube. Samples were immediately placed in liquid nitrogen after collection, transferred to the laboratory and stored at-80°C until DNA extraction.

### 2.2. Sample pretreatment and DNA extraction

For swab-collected samples (cage surface samples), the six-swab head was vortexed at maximum speed in a bead tube with sterile PBS. After discarding the swabs, the tube was centrifuged and the pellets were suspended in PowerBead solution and C1 buffer from the DNA isolation kit. Zirconium glass beads (400 mg; diameter, 0.1 mm) (BioSpec, Bartlesville, OK, United States) were added, and the mixture was shaken vigorously using an automatic rapid sample grinding instrument (JXFSTPRP-48, Shanghai Jingxin). The mixture was then incubated at 95°C for 5 min to maximize bacterial DNA extraction. All subsequent steps followed the manufacturer’s protocol for the PowerFecal DNA Kit (Qiagen, Germany). For DNA extraction with a PowerWater DNA Isolation Kit (Qiagen, Germany), the water samples and impinger liquid from the air samples were vacuum filtered onto sterile 0.22 μM polycarbonate filters (Sigma, St. Louis, MO, United States), transferred to 0.7 mm garnet bead tubes containing 1 mL of PW1 solution, and vortexed at maximum speed for 10 min. The remaining steps were performed according to the manufacturer’s protocol. To obtain microbial samples from the feather surface, the feather sample was divided into three parts and placed into a centrifuge tube containing 30 mL of PBS (0.1 mol/L, pH 7.0). After vortex oscillation at 180 r/min at 4°C for 3 h and ultrasonication at 4°C for 30 min, the feather sample cleaning solution was vacuum filtered onto sterile 0.22-μM polycarbonate filters (Sigma, St. Louis, MO, United States) and transferred to 0.7-mm garnet bead tubes containing 1 mL of PW1 solution. The mixture was shaken vigorously using a FastPrep-24 instrument, and the remaining steps were performed according to the manufacturer’s protocol. Uropygial glands (0.02 mg) were hydrolyzed with proteinase K before being processed with a DNA Mini Kit (Qiagen, Germany). Microbial DNA was extracted from the chicken feed using a DNeasy PowerFood Microbial Kit (Qiagen, Germany). DNA quality control was performed on the samples using several steps. Firstly, DNA degradation and potential contamination were monitored on 1% agarose gels. Secondly, DNA purity was assessed by determining the ratios of OD260/OD280 and OD260/OD230 using the NanoDrop ND-1000 Spectrophotometer (NanoDrop Technologies, Wilmington). Finally, DNA concentration was measured using the Qubit® dsDNA Assay Kit and Qubit 2.0 fluorometer (Invitrogen, Thermo Fisher Scientific, MA). DNA samples with OD values ranging from 1.8 to 2.0 and DNA contents above 1 μg were deemed suitable for library construction.

### 2.3. 16S rRNA sequencing

To analyze the microbiota community composition, specific V4 regions of the 16S rRNA gene were amplified with barcoded primers (F:5′-GTGCCAGCMGCCGCGGTAA-3′; R:5′-GGACTACHVGGGTWTCTAAT-3′) and PremixTaq (TaKaRa) was used for PCR amplification. PCR conditions were as follows: (1) 94°C for 3 min; (2) 30 cycles of 94°C for 30 s, 56°C for 30 s and 72°C for 30 s; and (3) a final extension at 72°C for 5 min. The amplicons were purified using a QIAquick PCR Purification Kit (Qiagen, Germany), and 250-bp read sequencing was performed on the Illumina HiSeq platform. The raw reads from 16S rRNA gene sequencing were demultiplexed and quality-filtered using Quantitative Insights into Microbial Ecology (QIIME2–2020.6) (Bokulich et al., 2013). Clean data were clustered using the Divisive Amplicon Denoising Algorithm 2 (DADA2) method with amplicon sequence variant (ASV) levels. The feature sequences were taxonomically assigned at the kingdom, phylum, class, order, family and genus levels. The α-diversity analysis, including the Chao1 and Shannon indexes, was conducted using QIIME2. For β-diversity analysis, the principal coordinates analysis (PCoA) was performed using QIIME2 to investigate the dissimilarities in bacterial community structure among samples. The 16S rRNA gene sequences in this study were deposited into the National Center for Biotechnology Information (NCBI) database (PRJNA855329).

### 2.4. Metagenomic sequencing and ARG analysis

The DNA extracted from the layer chicken cecal, feather, uropygial gland and cage samples on days 5, 30, and 57 for 16S rRNA gene sequencing was simultaneously used for metagenomic sequencing analysis. Sequencing libraries were generated with the NEBNext® Ultra™ DNA Library Prep Kit (E7645S, NEB, United States) according to the manufacturer’s recommendations. Then, the metagenomic libraries were sequenced on the Illumina HiSeq platform (Novogene, Beijing, China) using the 150-bp paired-end module. The raw reads were filtered and trimmed to obtain high-quality reads (clean reads), and quality control was performed using the following criteria: (1) reads with >10% unidentified nucleotides (*N*), (2) reads with ≥50% bases with mass fraction ≤20, and (3) reads aligned with barcodes were removed. The metagenomic sequences in this study were deposited into the NCBI database (PRJNA855329). We identified the taxonomic profiles (including kingdom, phylum, class, order, family, genus, and species information) of the metagenomic samples using kraken2. ARG-OAP v2.0 was applied to determine the ARG profiles (Yang et al., 2016). Briefly, potential ARG reads and 16S rRNA genes were extracted, and ARG-like reads were identified and annotated using the Perl package Ublastx_stageone X by combining the Comprehensive Antibiotic Resistance Database (CARD) and Antibiotic Resistance Gene Database (ARDB) (Yin et al., 2018). The normalized abundances of ARGs were expressed as “copies of ARGs per bacterial cell” (Jia et al., 2020). Species attribution analysis of resistance genes and resistance mechanism analysis were also conducted (McArthur et al., 2013; Jia et al., 2017).

### 2.5. SourceTracker

We used SourceTracker to evaluate the possible sources and proportions of microbial communities in the layer chicken cecum in the early stage. ASVs present in less than 1% of samples were first filtered, and the resultant ASV table was imputed with default parameters (Knights et al., 2011), with the layer chicken cecum at different days as the “sink” and the samples from different sources (feathers, uropygial gland, air, water, chicken feed and cage) identified as the “source.” The SourceTracker algorithm was then used to estimate the probability that the species in the intestinal samples came from the source environment (probability>80%).

To identify transfer events involving ARGs, SourceTracker was run with the default settings using the feather and cage ARGs as the source.

### 2.6. Statistical analysis

Data preparation was performed in Microsoft Excel 2019 (Microsoft, United States). SPSS 20.0 (IBM Corp, USA) was used to assess statistical significance, and the results were visualized with GraphPad Prism 8.0 software. The threshold for significance was set at *p* < 0.05. PCoA was performed using the coverage correlation matrix of the ARG subtypes. Venn diagrams were drawn with the Venn Diagram package. Network plots of the ARGs and bacterial communities (species level) were generated with Cytoscape 3.9.1 software.

## 3. Results

### 3.1. ARG distribution characteristics and differences among layer chicken cecal, feather, and cage samples

**Figure 1:**
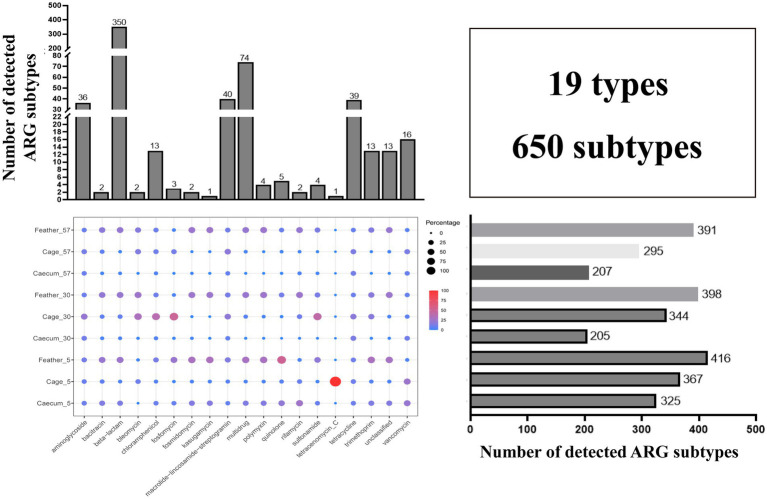
Statistics of ARG diversity between groups. The total number of detected ARG subtypes in different ARG types in all samples is shown on top, the total number of detected ARG subtypes in different groups is shown on the right, and the bubble chart shows the relative abundance of each ARG type in different groups.

**Figure 2:**
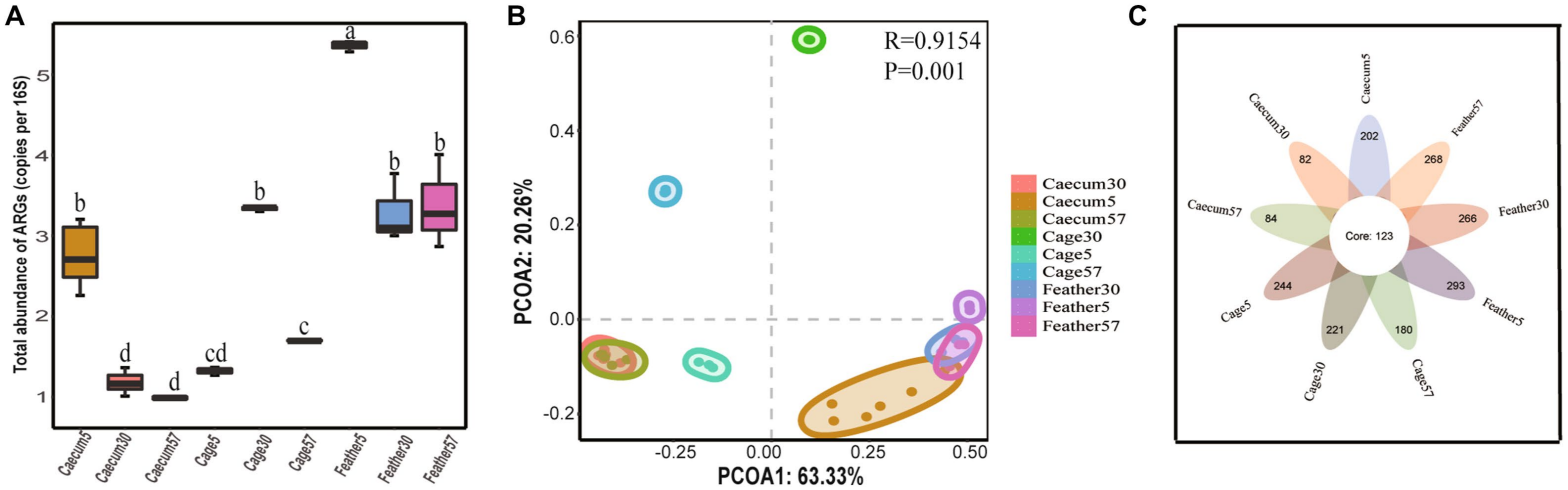
ARG distribution characteristics. **(A)** The total abundance of ARGs in different groups. In the box chart, “□” represent the means, boxes represent the upper and lower quartiles, lines represent the medians, and whiskers represent ranges. **(B)** PCoA plots showing the ARG composition differences among groups. **(C)** Venn diagram showing the number of shared and unique ARGs among groups.

A total of 620 ARG subtypes were detected in the layer chicken cecal, feather and cage samples, even though no antibiotics were used on the farm. These ARGs were associated with 19 antibiotic types (350 β-lactam, 74 multidrug, 40 macrolide-lincosamide-streptogramin (MLS), 39 tetracycline, and 36 aminoglycoside resistance genes, among others) (Figure 1). The number of ARG subtypes in each group varied, ranging from 205 to 416. The 10 most abundant ARG subtypes in the different groups are summarized in Supplementary Table S1. The feathers on day 5 contained the most ARG types (416), and the total abundance was also the highest. The layer chicken cecum contained the fewest types (205), and there was a significant decrease in the total abundance of ARGs in the chick cecum over time (Figure 2A). These changes may be related to changes in gut microbes. Notably, no resistance genes were detected in the uropygial gland microbiome samples.

We then assessed the global similarity of ARG composition in each group based on principal coordinates analysis (PCoA). The results showed that the layer chicken cecum on day 5 differed from that on day 30 and day 57 in terms of ARGs, and the cage ARGs from different days clustered together, indicating that the ARGs composition in different groups changed over time. In addition, the results showed that the layer chicken cecum ARGs on day 5 were overall more similar to the feather ARGs than to the cage ARGs (Figure 2B), implying that the feather ARGs might have been transmitted to the layer chicken cecum on day 5.

A total of 123 ARGs belonging to 17 types were shared by the layer chicken cecal, feather and cage samples on different days (Figure 2C, summarized in Supplementary Table S2). In terms of total coverage, these 123 shared ARGs contributed to 97.01 ± 0.79%, 95.13 ± 0.08%, and 91.15 ± 0.10% of the total ARGs detected in the chick cecal and feather samples on day 5; 84.80 ± 3.4%, 63.17 ± 0.29%, and 93.30 ± 0.83% on day 30; and 82.2 ± 4.3%, 67.89 ± 0.36%, and 94.34 ± 0.17% on day 57 (Supplementary Figure S2). These shared ARGs included 34 multidrug resistance genes (*emrA*, *emrB*, *mdtA*, *TolC*, etc.) and 13 tetracycline resistance genes (*tetA*, *tetW*, *totO*, *tet44*, etc.). There were more unique ARGs in the cage and feather samples than in the layer chicken cecal samples.

### 3.2. Microbial community composition in different microbial sources

Supplementary Figure S3 shows the alpha diversity of different groups. Both the richness index and Shannon index revealed an increase in microbial diversity in the layer chicken cecum over time, and there was a significant difference in alpha diversity among the microbial sample types from different sources (uropygial gland>cage>feather; *p* < 0.05).

**Figure 3:**
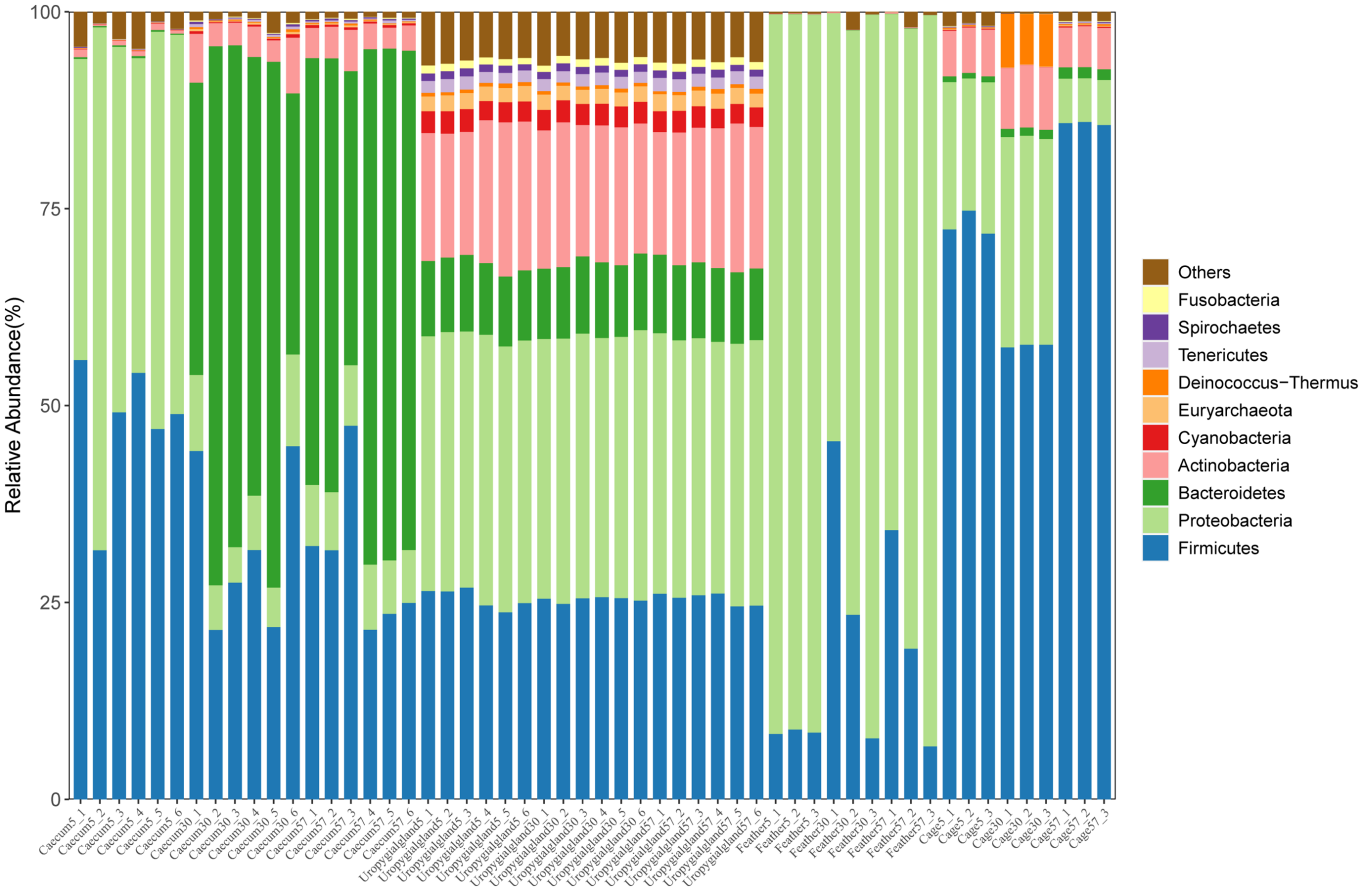
Relative abundances of the top 10 phyla in the layer chicken cecal, feather, uropygial gland and cage samples.

The results showed that Proteobacteria (48.31 ± 9.18%) and Firmicutes (47.32 ± 7.62%) were the major phyla in the layer chicken cecum on day 5 (Figure 3); at the genus level, they were dominated by *Escherichia* (36.83 ± 8.53%) and *Flavonifractor* (15.44 ± 4.61%) (Supplementary Figure S4). Similarly, increased relative abundances of Bacteroidetes and Actinobacteria and a decreased relative abundance of Proteobacteria were observed in the layer chicken cecum (*p* < 0.05) on day 30 and day 57. *Bacteroides* and *Alistipes* were the major genera in the layer chicken cecum on days 30 and 57. Furthermore, among the different microbial sources, Proteobacteria, Firmicutes and Actinobacteria were the major phyla in the uropygial gland samples, Firmicutes were dominated by *Escherichia* in the feather samples, and Firmicutes were dominated by *Lactobacillus* in the cage samples.

Further analysis showed shared and unique bacteria among the layer chicken cecal, feather, uropygial gland and cage samples (Supplementary Figure S5). The results showed that between days 5 and 57, the number of microbial species increased from 2,998 to 4,708, indicating that the gut microbes early in life are derived from a dramatic and complex transition from a near-sterile state to extremely dense colonization. More bacterial species (2885) in the layer chicken cecum were shared with the uropygial gland than with the other groups on day 5. At days 30 and 57, the layer chicken cecum shared a high number (2,869 and 3,379 respectively) of microbial species with the cage samples, and some opportunistic pathogens, such as *Escherichia* and *Enterococcus* species, were also shared among the cage, feather, uropygial gland and layer chicken cecal samples.

### 3.3. Layer chicken microbial sources and estimated proportions of the early gut microbial communities

**Figure 4:**
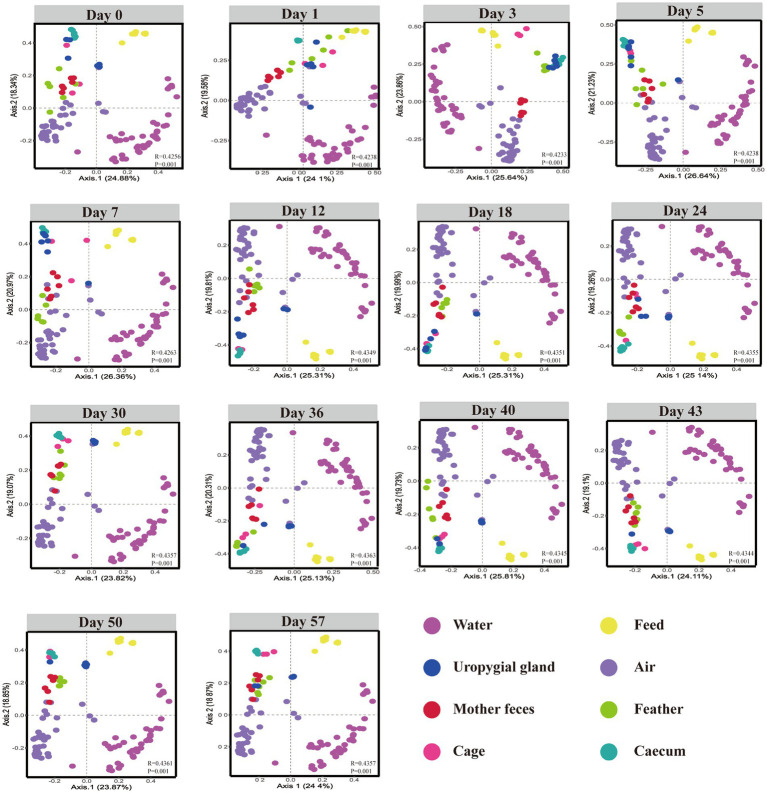
β-Diversity of the cecal, feed, maternal fecal, uropygial gland, feather, water, air and cage samples. Bray–Curtis dissimilarity was calculated using the abundance of ASVs.

To investigate the development and potential sources of gut microbiota in layer chickens, we collected cecal contents and corresponding environmental samples, including feather, uropygial gland, water, air, feed, and cage microbiota samples at post-hatching days 0, 1, 3, 5, 7, 12, 18, 24, 30, 36, 40, 43, 50, and 57. The PCoA ordination based on Bray–Curtis dissimilarity showed that the early layer chicken cecal samples (at days 0 and 7) clustered with the layer chicken uropygial gland and feather samples, but they gradually diverged with age, eventually showing some similarities with the cage sample (Figure 4). These results suggest that the uropygial gland, feather, and cage microbiomes may play a role in the early colonization of the cecal microbiome in layer chickens, but further investigation is needed to determine the extent and mechanisms of transmission.

**Figure 5:**
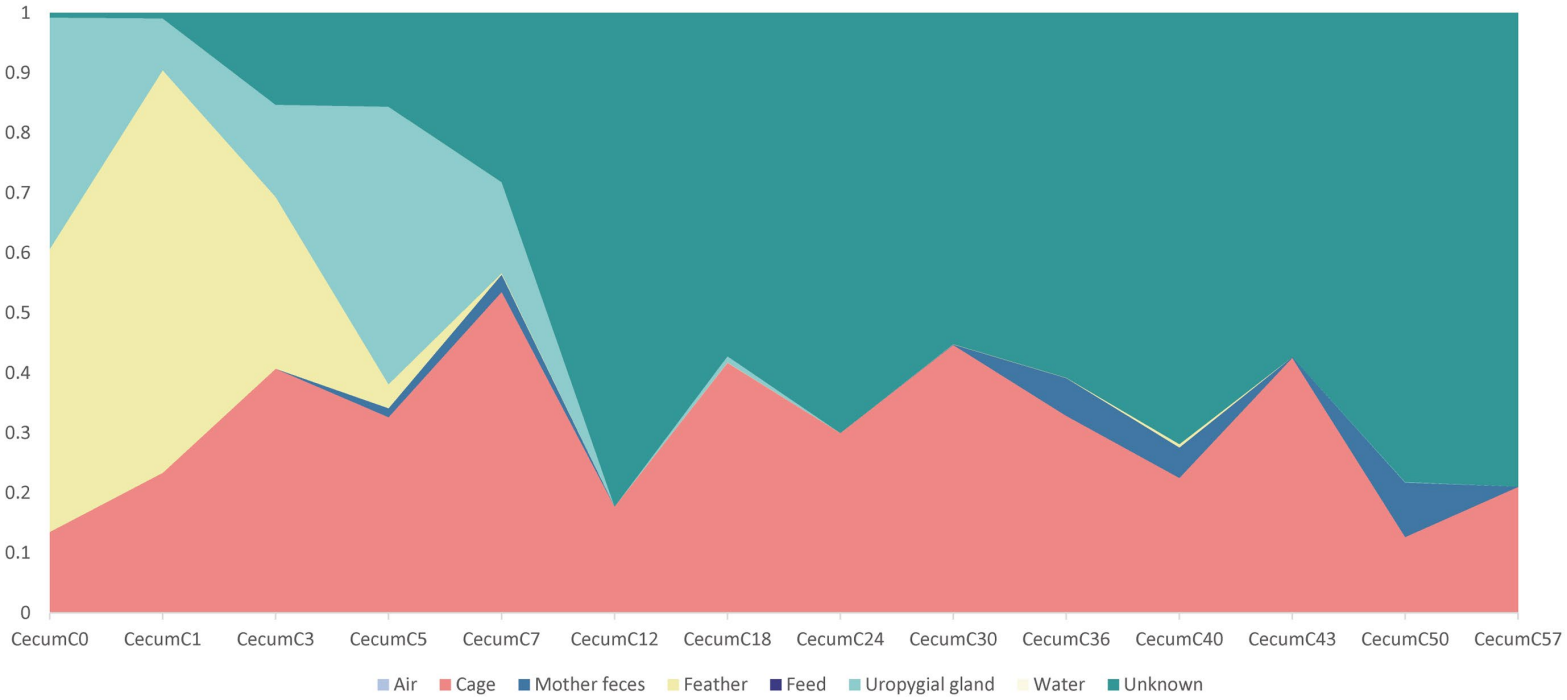
Dynamic contributions of different microbial sources to the layer chickens cecal microbiota during the first 57 days. The proportion of the microbiota from cecal samples of layer chicken was estimated as originating from different sources (colored regions) using bacterial source tracking.

SourceTracker, a Bayesian probability tool, was used to further investigate how different microbial sources contributed to the cecal community assembly of hatchlings. The results revealed that the feather and uropygial gland microbiota contributed the most to the cecal (day 0) microorganisms compared with other sources, with contributions of 47.0 and 38.6%, respectively, but their contributions gradually declined with age. Interestingly, the relative contribution of the cage microbiota to the chick cecal microbiome was more than 12% at all stages, indicating that the cage may be the primary environmental source of bacterial communities in layer chickens, especially seven days after hatching (Figure 5). To our surprise, there was little evidence that water and air microbes colonized hatchlings that had a microbiota from largely unknown sources.

Next, we sought to identify specific genera transmitted from different microbial sources to the layer chicken cecum. These transmission events included diverse taxa from Firmicutes, Proteobacteria and Bacteroidetes, some of which (*Escherichia*, *Enterococcus*, *Ruminococcaceae*, and *Helicobacter*) include opportunistic pathogenic strains responsible for zoonotic infections (Supplementary Figure S6). Among them, *Escherichia* was the main bacterial taxon transmitted from feathers to the layer chicken cecum at day 0, with a contribution rate of 40.8%, and *Ruminococcaceae* was mainly transmitted to the cecum through the cage, with a contribution rate of 3.0%. The transmission of these opportunistic pathogenic strains indicates that the surroundings during feeding may pose underappreciated occupational hazards in industrialized farming. To confirm that the species and their resistance genes were environmentally acquired, we performed metagenomic analysis of the layer chicken cecal, feather, uropygial gland and cage microbiome samples at days 5, 30, and 57.

### 3.4. Transmission of microbes and ARGs from cages and feathers to the layer chicken cecum

**Figure 6:**
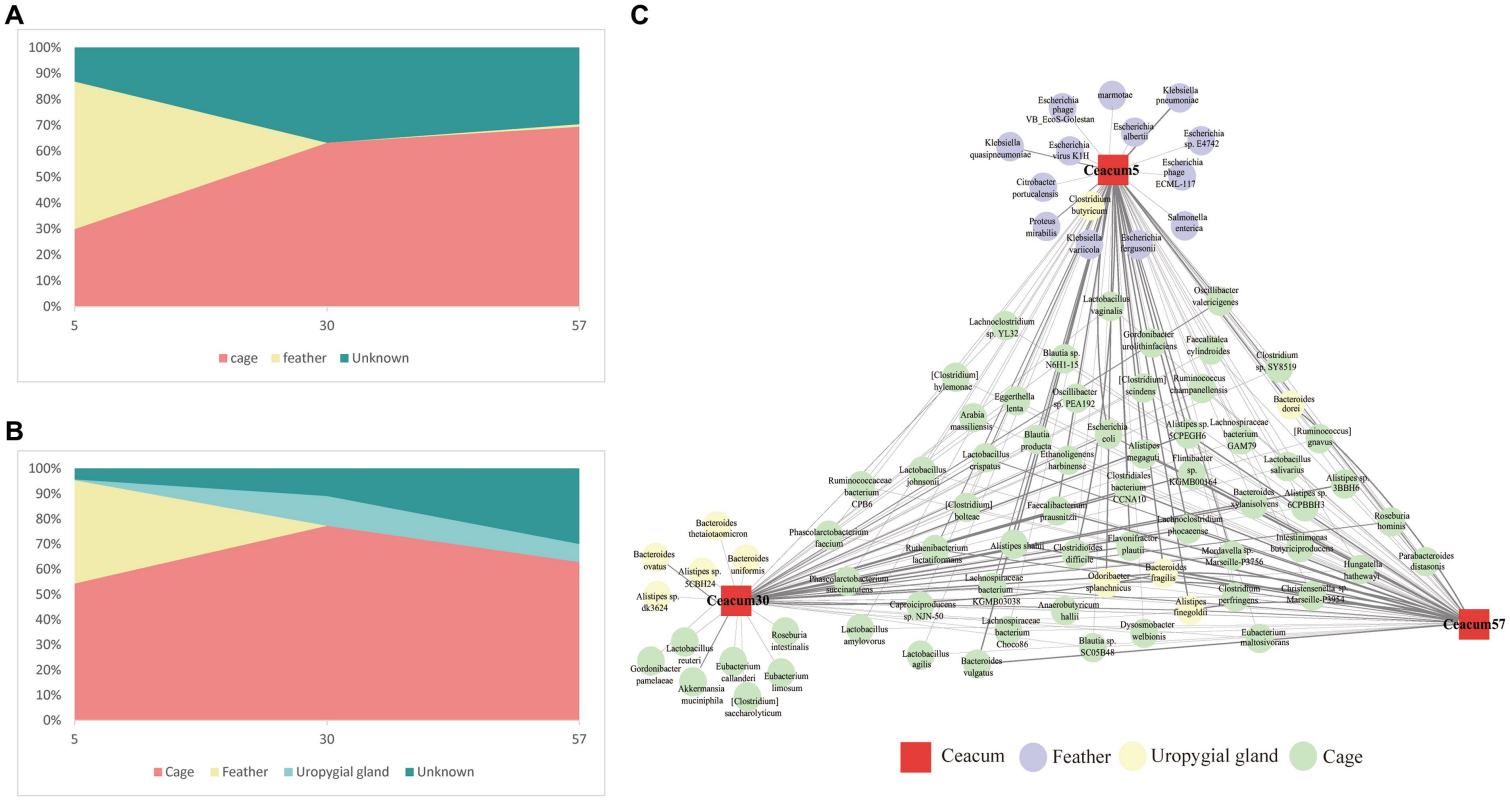
Transmission of microbes and ARGs from feathers and cages to the layer chicken cecum. **(A)** Predicted proportions of ARGs in the layer chicken gut microbiota at different days after hatching (5, 30, and 57) that originated from their feathers and cages. **(B)** Proportion of the microbiota from the cecal samples of layer chickens at different days estimated as having originated from the uropygial gland, feather and cage. **(C)** Microbial species transmission network from the feather and cage to the layer chicken cecum. The red square depicts the intestine (different days are displayed in the center of each node), circles indicate transmitted species, connecting arrows represent the transmission events, and the edge thickness is equivalent to the magnitude of the contribution.

The results of the SourceTracker algorithm analysis showed that, on day 5, 30% of the layer chicken cecal ARGs were from the cage, 57% were from feathers and 13% were from unknown sources. The contribution of cage ARGs became progressively more prominent with age, and cage ARGs contributed 63.3 and 69.5% of the layer chicken cecal ARGs between days 30 and 57 (Figure 6A). In addition, we identified 290 ARGs involved in transfer from cage and feather samples to the layer chicken cecum, including 152 β-lactam resistance genes, 45 multidrug resistance genes, 21 tetracycline resistance genes and 17 MLS resistance genes. Among them, 240 ARGs contributed more than 0.1% (summarized in Supplementary Table S3).

Members of the bacterial community are the main hosts of ARGs. To identify the ARG hosts more accurately, metagenomics was used to analyze the species attributions of ARGs. A total of 4,364 species were the main 240 ARG hosts. However, not all ARG-carrying bacteria can be transmitted from cages and feathers to the chick cecum. The SourceTracker algorithm was again used to assess bacterial transmission from the cage and feather to the layer chicken cecal samples (Figure 6B). The results showed that on day 5, 54.4% of the layer chicken cecal microbes originated from cages, 41% from feathers, 0.32% from the uropygial gland and 4.2% from unknown sources. The contribution of cage microbes gradually increased to 77.3% on day 30, and that of the uropygial gland microbes to cecal microbes increased to 11.8%, while the contribution of feathers to the cecum decreased sharply and was negligible. At day 57, the contribution of the cage microbes to cecal microbes decreased slightly to 63%, and the contribution of the uropygial gland was 7.1%. A ternary plot was used to more intuitively reflect the contribution of various bacterial sources to each cecal microbiome of layer chickens on different days. As shown in the plot, at day 5, almost all the layer chicken samples showed a uniform distribution between feather and cage samples, and the layer chicken cecal samples gradually became increasingly closely related to the cage samples over time (Supplementary Figure S7), which was similar to the results of the ARG source analysis.

Among them, we identified 85 species that contributed more than 0.1% (Figure 6C). The cage contributed the greatest number of bacterial species (57) on day 5, and the highest contributions were *Bacteroides fragilis* (11.5%), *Bacteroides dorei* (6.5%) and *Odoribacter splanchnicus* (4.8%). The feathers contributed 15 kinds of bacteria, and the highest contributions were *Escherichia coli* (32.0%) and *Klebsiella pneumoniae* (3.4%). The uropygial gland contributed only one bacterium, *Clostridium butyricum*, and the contribution was low (0.12%). On day 30, the cage contributed the highest number of bacterial species (56), including *B. fragilis* (38.0%), *Flavonifractor plautii* (3.2%) and *Clostridiales bacterium CCNA10* (3.0%), and the uropygial gland contributed 8 bacterial species, including *B. fragilis* (3.1%) and *Bacteroides ovatus* (0.6%). On day 57, the number of bacterial species contributed by the cage increased to 59, including *B. fragilis* (11.5%), *B. dorei* (6.5%), *O. splanchnicus* (4.8%) and *Faecalibacterium prausnitzii* (3.1%). The number of bacterial species contributed by the uropygial gland decreased to 4, namely, *B. fragilis* (1.1%), *Alistipes finegoldii* (0.6%), *O. splanchnicus* (0.5%) and *B. dorei* (0.4%).

**Figure 7:**
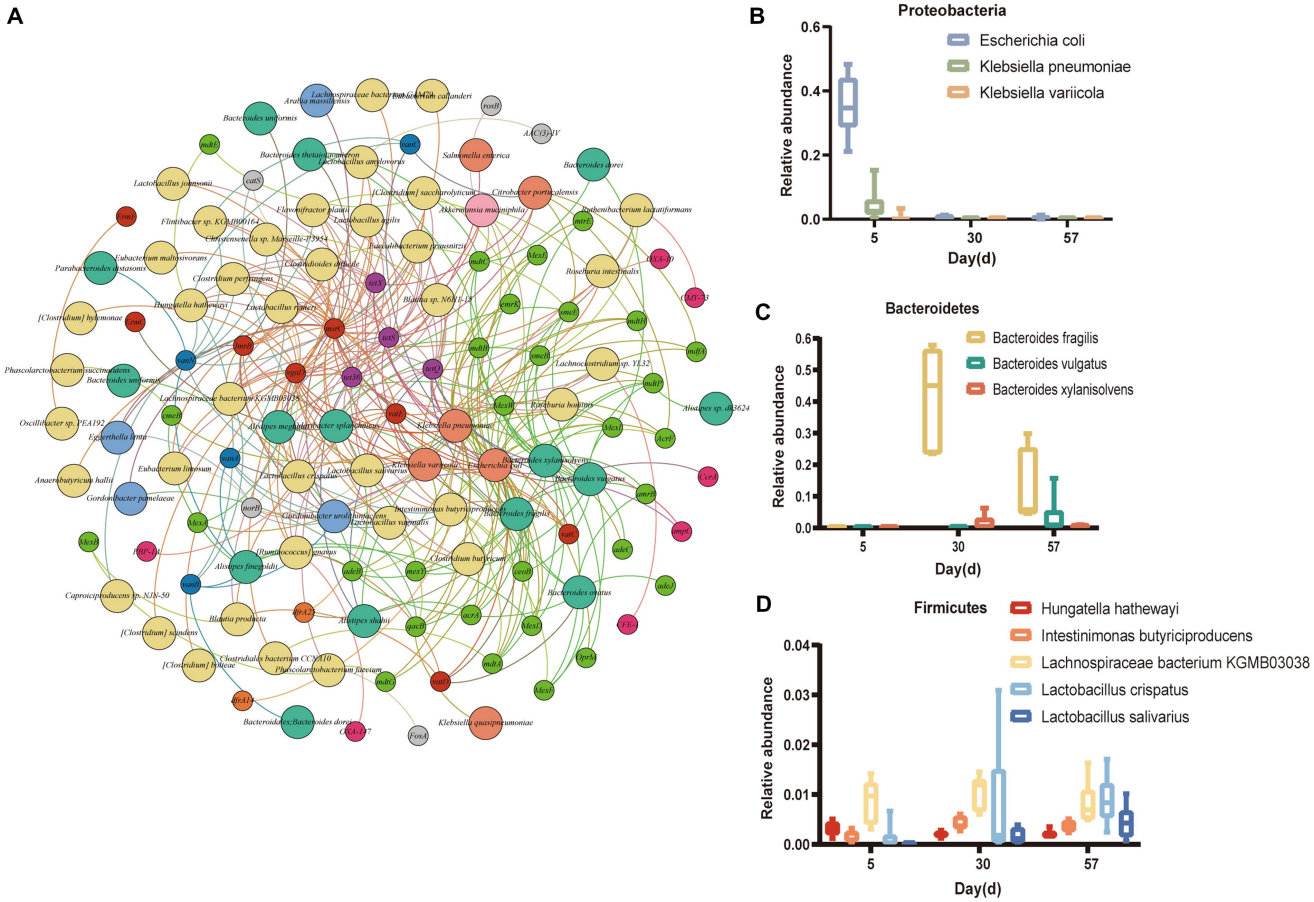
Microbial (species-level) attributions of ARGs. **(A)** The connecting line between two points indicates an affiliation. For example, emrk is linked to *Escherichia coli*, indicating that *Escherichia coli* contained *emrk*. The large nodes represent bacterial phyla, and the small nodes represent ARG types. The nodes are colored in accordance with the ARG types or phyla. **(B-D)** Relative abundances of the top 11 ARG-carrying bacteria with the largest number of ARG type variations over time [Proteobacteria **(B)**, Bacteroidetes **(C)** and Firmicutes **(D)**].

Next, we analyzed the correlations between 85 bacterial species and 240 ARGs, and the results indicated that 63 bacterial species were speculated to be possible hosts of 60 ARGs (Figure 7A). For instance, *K. pneumoniae* was a potential host for 32 ARG subtypes, 23 of which were multidrug resistance genes (*acrA*, *emrK*, *mdfA*, *mdtB*, *mexA*, *mtrE*, etc.), 3 were tetracycline resistance genes (*tetQ*, *tetS* and *tetX*), 3 were MLS resistance genes (*lmrB*, *mscrC* and *vgaD*), 2 were vancomycin resistance genes (*vanA* and *vanC*) and 1 was a quinolone resistance gene (*norB*). *E. coli* was a potential host for 24 ARG subtypes, 16 of which were multidrug resistance genes (*acrA*, *emrK*, *mdtA*, etc.), 3 were MLS resistance genes (*msrC*, *vatD*, *vatE* and *vgaD*), 2 were tetracycline resistance genes (*tetQ* and *tetX*), 1 was a β-lactam resistance gene (*ampC*) and 1 was a vancomycin resistance gene (*vatC*). *B. fragilis* was a potential host for 13 ARG subtypes, such as *acrA* and *emrB*. In addition, *Intestinimonas butyriciproducens* and *Clostridioides difficile* were the predominant hosts and harbored most of the diverse ARG subtypes for multidrug resistance. Figures 7B-D shows the 11 bacteria with the highest number of resistance gene types. The results show that the relative abundances of *K. pneumoniae* and *E. coli* gradually decreased, but those of *L. salivarius L. bacterium KGMB03038* gradually increased. These results suggest an extensive exchange of antibiotic-resistant bacteria between layer chickens and their surrounding environments.

## 4. Discussion

By the metagenomic shotgun sequencing method, we identified 650 ARGs across layer chicken cecal, feather and cage samples on days 5, 30, and 57, and more diverse ARGs were observed in the cage and feather samples than in the layer chicken cecal sample. A total of 123 ARGs were shared among all samples, and these were mainly multidrug resistance genes and tetracycline resistance genes. The contribution rates of 123 shared ARGs to the gut ARGs of layer chickens were all more than 82%, suggesting that the environment is an important contributor to the gut ARGs in layer chickens (Ding et al., 2022).

The number and total abundance of gut ARGs in layer chicken decreased significantly over time, consistent with previous studies showing that the fecal ARG abundance in the brooding period was higher than that in the growing period (Zhu et al., 2021). The microbial community was the key factor that directly affected the ARGs (Wang et al., 2020). We identified ARG hosts using metagenomic assembly-based host-tracking analysis. The results showed that *K. pneumoniae*, *E. coli*, *I. butyriciproducens* and *C. difficile* were the main host bacteria of ARGs. The relative abundance of these ARG-carrying bacteria gradually decreased in the layer chickens, which may explain the decrease in total ARG abundance. It has been suggested that *K. pneumoniae* has a flexible ability to accumulate and switch resistance and is the host of many ARGs; together with other highly important multidrug-resistant pathogens, it has been classified as an ESKAPE organism (Navon-Venezia et al., 2017). In our study, we found that *K. pneumoniae* was the host of 32 ARGs. In addition, *E. coli* is also a common host of ARGs; antibiotic-resistant *E. coli* strains carrying different ARGs have been widely studied as possible environmental pollutants in recent years, and their dissemination poses potential risks to human health (Power et al., 2016; Chika et al., 2019; Zhang et al., 2020). We found that *E. coli* harbored 24 ARGs, including *acrA*, *tetX*, and *lmrB*. Zhou et al. (2020) found that the poultry microbial community shifts after antibiotic administration were mainly induced by increased abundances of the families *Escherichia/Shigella* and *Klebsiella*. However, no antibiotics were used during the whole experiment, and the relative abundance of *Escherichia* peaked at day 5, which was gradually replaced by the probiotic *Bacteroides.* This suggests that banning the use of antibiotics plays a positive role in maintaining gut microbial homeostasis and reducing ARG levels in laying hens. It is worth noting that this study found that probiotics (e.g., *B. fragilis*) are also potential hosts. It has been shown that polysaccharide A (PSA) of *B. fragilis* is the archetypical example of a commensal molecule that can modulate the host immune system in health and disease (Erturk-Hasdemir and Kasper, 2018). In this experiment, we found that *B. fragilis* is the host for *tetQ* and *tetS*. This suggests that probiotics may contain ARGs. Feeding with probiotics carrying a high abundance of ARGs may further increase the risk of ARG transmission in the environment.

Furthermore, through SourceTracker analysis of the sources of related ARGs and microbes, it was found that the environment contributed more to the shaping of the gut microbiota, and the contribution of adult hen to the layer chickens only 2.5% at day7.However the cecal microbiota of chicks remaining in contact with an adult hen developed quickly and within a week reached a composition similar to that observed in adult hen (Kubasova et al., 2019). This was mainly because in commercial laying hen production, the microbes attached to the surface of the eggshell are killed because of the treatment of eggs (disinfection and fumigation) before hatching (Videnska et al., 2014). Direct contact between chicks and parents after hatching does not occur as in wild birds, so the external environment plays a vital role in shaping the gut microbial community in layer chickens (Maki et al., 2020). In this study, beta and SourceTracker analysis revealed that layer chicken gut microbes were more similar to feather and uropygial gland microbes after hatching, which has not been reported previously. We speculate that this may be related to the commercial production mode, in which layer chickens that hatch at the same time are usually fed together and are afraid of the cold, easily frightened and have a clustering habit (Appleby et al., 2004). In addition, compared with free layer chickens, caged layer chickens feed daily due to their appetite, so the nutritional needs of layer chickens can be satisfied sooner, while the feeding density is large and the activity space is restricted, thus causing layer chicken psychological tension and anxiety, which can easily induce feather pecking and anal pecking (Tahamtani et al., 2022). Therefore, feather and uropygial gland microbes, including *E. coli*, *K. pneumoniae* and *C. butyricum*, are more likely to be transmitted to the gut for colonization. Among them, *K. pneumoniae* and *E. coli* are the main hosts of ARGs. It is worth noting that the SourceTracker results of the ARGs were similar to those of microorganisms. Early ARGs came mainly from feathers. These results suggest that the early gut ARGs in layer chickens may originate *via* the transmission of microbes from feathers.

However, cage microbes gradually became the major source of gut microbes in layer chickens. Although the cage was cleaned and disinfected before raising chickens, the frequent activities of layer chickens in the cage created a unique microbial environment. Studies have shown that feather pecking is negatively correlated with ground pecking and that a decrease in feather pecking is associated with an increase in ground pecking (Aneja et al., 2008). Frequent ground pecking in poultry leads to horizontal transmission of microbes, and cage microbes may originate from the feces of layer chickens, dust in the air, water and feed residues. However, the contribution of air and water bacteria to the colonization of the layer chicken cecum was very low in our study. The reason for this may be that the aerobic bacteria in the air did not adapt well to the anaerobic environment of the layer chicken gut. Volf et al. (2021) found that the feed microbiota and water microbiota were not the major sources of gut anaerobes for chickens in commercial production. Litter microbes have a great influence on poultry gut microbes in commercial production (Wang et al., 2016). Cressman et al. (2010) showed the cycling of certain bacteria between the litter and gut of poultry. The same results were found in commercially produced lambs and piglets (Bi et al., 2019; Chen et al., 2020). The results showed that the early gut was more easily colonized by cage microbes when the chicks were not in contact with their parents. The frequent exchange of ARG-carrying bacteria between the gut and cage of layer chickens may lead to the accumulation of ARGs in the gut and the environment, posing a threat to animal and environmental safety.

Although, in this study, we carried out comprehensive sampling of commercial layer chickens, the samples were used for SourceTracker analysis of gut microbes and ARGs in layer chickens, and the study still had several limitations. For example, since the experimental site was a brood farm, samples could not be collected during the laying period for a more comprehensive and systematic assessment of microbial and ARG transmission.

Despite these shortcomings, we can still provide some suggested management strategies for layer chickens. In daily management, (1) attention should be given to reducing the stimulation of layer chickens and reducing rearing density to reduce the feather pecking and anal pecking behavior of layer chickens. (2) The cages should be kept clean and hygienic, and any remaining feces and food residues in the cages should be promptly removed. This will not only reduce the transmission of ARGs and ARG host bacteria from the environment to the layer chicken gut but also help improve animal welfare.

## 5. Conclusion

There was a high relative abundance of ARGs in the layer chicken cecal, feather and cage samples, including ARGs that confer resistance to multiple drugs, such as tetracycline, aminoglycosides, and β-lactams. The layer chicken cecal ARGs originated mainly from cage. The variations in ARG profiles in layer chicken cecal samples and identified the bacterial species that primarily influence these changes, including *K. pneumoniae*, *E. coli*, *B. fragilis*, and *B. dorei* Our findings indicate that these bacteria are mainly transmitted from the cage to the layer chicken cecum. The cycling of ARG-carrying bacteria between these two environments may result in the accumulation of ARGs in the gut and cage environment, ultimately posing a potential risk to animal health. It is noteworthy that *B. fragilis* is an opportunistic pathogen that may cause diarrhea in laying hens, suggesting that the transmission of opportunistic pathogens may pose a dual risk. However, further experiments are needed to verify this risk. These results can provide reference data for the healthy breeding of layer chickens and the prevention and control of ARG pollution.

## Data availability statement

The data presented in the study are deposited in the National Center for Biotechnology Information (NCBI) repository, accession number PRJNA855329 can be found in the article/Supplementary material.

## Ethics statement

The present study followed the institutional guidelines for the care and use of animals, and all experimental procedures involving animals were approved by the Animal Experimental Committee of South China Agricultural University (Ethics Approval Code: SYXK 2014–0136).

## Author contributions

SX: conceptualization, data curation, formal analysis, methodology, writing—original draft, and writing—review and editing. JM: data curation, conceptualization, methodology, and supervision. YC: conceptualization, investigation, methodology, formal analysis, and data curation. KF and LM: methodology, data curation. XL and YW: conceptualization and supervision. Yan Wang: conceptualization, investigation, writing—review and editing, resources, supervision, and funding. All authors contributed to the article and approved the submitted version.

## Funding

This research was funded by the National Natural Science Foundation of China (31972610). The Construction Project of Modern Agricultural Science and Technology Innovation Alliance in Guangdong Province (2022KJ128 and 2023KJ128). The Guangdong agricultural research projects (YUECAINONG202137). The Heyuan Branch, Guangdong Laboratory for Lingnan Modern Agriculture Project (DT20220020). The earmarked fund for Modern Agro-industry Technology Research System (CARS-40).

## Conflict of interest

The authors declare that the research was conducted in the absence of any commercial or financial relationships that could be construed as a potential conflict of interest.

## Publisher’s note

All claims expressed in this article are solely those of the authors and do not necessarily represent those of their affiliated organizations, or those of the publisher, the editors and the reviewers. Any product that may be evaluated in this article, or claim that may be made by its manufacturer, is not guaranteed or endorsed by the publisher.

## References

[ref1] Aneja V. P. Blunden J. Roelle P. A. Schlesinger W. H. Knighton R. Niyogi D.. (2008). Workshop on agricultural air quality: state of the science. Atmos. Environ. 42, 3195–3208. doi: 10.1016/j.atmosenv.2007.07.043, PMID: 37138143

[ref2] Appleby M. C. Mench J. A. Hughes B. O. (2004). Poultry behaviour and welfare. Cambridge: CABI Publishing.

[ref3] Bi Y. Cox M. S. Zhang F. Suen G. Zhang N. Tu Y.. (2019). Feeding modes shape the acquisition and structure of the initial gut microbiota in newborn lambs. Environ. Microbiol. 21, 2333–2346. doi: 10.1111/1462-2920.14614, PMID: 30938032 PMC6849743

[ref4] Bokulich N. Subramanian S. Faith J. Gevers D. Gordon J. Knight R.. (2013). Quality-filtering vastly improves diversity estimates from Illumina amplicon sequencing. Nat. Methods 10, 57–59. doi: 10.1038/nmeth.2276, PMID: 23202435 PMC3531572

[ref5] Chen C. Chen C. Chen Y. Fang A. Shaw G. Hung C.. (2020). Maternal gut microbes shape the early-life assembly of gut microbiota in passerine chicks via nests. Microbiome 8, 1–13. doi: 10.1186/s40168-020-00896-9 32917256 PMC7488855

[ref6] Chika F. Odum O. N. (2019). Freshwater environments as reservoirs of antibiotic resistant bacteria and their role in the dissemination of antibiotic resistance genes. Environ. Pollut. 254:113067. doi: 10.1016/j.envpol.2019.113067 31465907

[ref7] Chin C. Y. Tipton K. A. Farokhyfar M. Burd E. M. Weiss D. S. Rather P. N. (2018). A high-frequency phenotypic switch links bacterial virulence and environmental survival in *Acinetobacter baumannii*. Nat. Microbiol. 3, 563–569. doi: 10.1038/s41564-018-0151-5, PMID: 29693659 PMC5921939

[ref8] Cressman M. D. Yu Z. Nelson M. C. Moeller S. J. Lilburn M. S. Zerby H. N. (2010). Interrelations between the microbiotas in the litter and in the intestines of commercial broiler chickens. Appl. Environ. Microbiol. 76, 6572–6582. doi: 10.1128/AEM.00180-10, PMID: 20693454 PMC2950482

[ref9] Diaz Carrasco J. M. Casanova N. A. Fernández Miyakawa M. E. (2019). Microbiota, gut health and chicken productivity: what is the connection? Microorganisms. 7:374. doi: 10.3390/microorganisms7100374, PMID: 31547108 PMC6843312

[ref10] Ding J. Dai R. Yang L. He C. Xu K. Liu S.. (2017). Inheritance and establishment of gut microbiota in chickens. Front. Microbiol. 8:1967. doi: 10.3389/fmicb.2017.01967 29067020 PMC5641346

[ref11] Ding D. Zhu J. Gao Y. Yang F. Ma Y. Cheng X.. (2022). Effect of cattle farm exposure on oropharyngeal and gut microbial communities and antibiotic resistance genes in workers. Sci. Total Environ. 806:150685. doi: 10.1016/j.scitotenv.2021.150685, PMID: 34600986

[ref12] Erturk-Hasdemir D. Kasper D. L. (2018). Finding a needle in a haystack: *Bacteroides fragilis* polysaccharide a as the archetypical symbiosis factor. Ann. N. Y. Acad. Sci. 1417, 116–129. doi: 10.1111/nyas.13660, PMID: 29528123

[ref13] He L.-Y. Liu Y.-S. Su H.-C. Zhao J.-L. Liu S.-S. Chen J.. (2014). Dissemination of antibiotic resistance genes in representative broiler feedlots environments: identification of indicator ARGs and correlations with environmental variables. Environ. Sci. Technol. 48, 13120–13129. doi: 10.1021/es5041267, PMID: 25338275

[ref14] Hu Y. Cheng H. (2016). Health risk from veterinary antimicrobial use in China's food animal production and its reduction. Environ. Pollut. 219, 993–997. doi: 10.1016/j.envpol.2016.04.099, PMID: 27180067

[ref15] Jia S. Bian K. Shi P. Ye L. Liu C. H. (2020). Metagenomic profiling of antibiotic resistance genes and their associations with bacterial community during multiple disinfection regimes in a full-scale drinking water treatment plant. Water Res. 176:115721. doi: 10.1016/j.watres.2020.115721, PMID: 32222544

[ref16] Jia B. Raphenya A. Alcock B. Waglechner N. Guo P. Tsang K.. (2017). CARD 2017: expansion and model-centric curation of the comprehensive antibiotic resistance database. Nucleic Acids Res. 45, D566–D573. doi: 10.1093/nar/gkw1004, PMID: 27789705 PMC5210516

[ref17] Knights D. Kuczynski J. Charlson E. Zaneveld J. Mozer M. Collman R.. (2011). Bayesian community-wide culture-independent microbial source tracking. Nat. Methods 8, 761–763. doi: 10.1038/nmeth.1650, PMID: 21765408 PMC3791591

[ref18] Kubasova T. Kollarcikova M. Crhanova M. Karasova D. Cejkova D. Sebkova A.. (2019). Contact with adult hen affects development of caecal microbiota in newly hatched chicks. PLoS One 14:e0212446. doi: 10.1371/journal.pone.0212446, PMID: 30840648 PMC6402632

[ref19] Lee S. La T.-M. Lee H.-J. Choi I.-S. Song C.-S. Park S.-Y.. (2019). Characterization of microbial communities in the chicken oviduct and the origin of chicken embryo gut microbiota. Sci. Rep. 9, 1–11. doi: 10.1038/s41598-019-43280-w 31048728 PMC6497628

[ref20] Li X. Stokholm J. Brejnrod A. Vestergaard G. A. Russel J. Trivedi U.. (2021). The infant gut resistome associates with *E. coli*, environmental exposures, gut microbiome maturity, and asthma-associated bacterial composition. Cell Host Microbe 29:e974, 975–987. doi: 10.1016/j.chom.2021.03.017 33887206

[ref21] Liu H. Zeng X. Zhang G. Hou C. Li N. Yu H.. (2019). Maternal milk and fecal microbes guide the spatiotemporal development of mucosa-associated microbiota and barrier function in the porcine neonatal gut. BMC Biol. 17, 1–15. doi: 10.1186/s12915-019-0729-2 31852478 PMC6921401

[ref22] Maki J. J. Bobeck E. A. Sylte M. J. Looft T. (2020). Eggshell and environmental bacteria contribute to the intestinal microbiota of growing chickens. J. Anim. Sci. Biotechnol. 11, 1–17. doi: 10.1186/s40104-020-00459-w 32537141 PMC7288515

[ref23] McArthur A. G. Waglechner N. Nizam F. Yan A. Azad M. A. Baylay A. J.. (2013). The comprehensive antibiotic resistance database. Antimicrob. Agents Chemother. 57, 3348–3357. doi: 10.1128/AAC.00419-13, PMID: 23650175 PMC3697360

[ref24] Navon-Venezia S. Kondratyeva K. Carattoli A. (2017). *Klebsiella pneumoniae*: a major worldwide source and shuttle for antibiotic resistance. FEMS Microbiol. Rev. 41, 252–275. doi: 10.1093/femsre/fux013, PMID: 28521338

[ref26] Power M. L. Samuel A. Smith J. J. Stark J. S. Gillings M. R. Gordon D. M. (2016). *Escherichia coli* out in the cold: dissemination of human-derived bacteria into the Antarctic microbiome. Environ. Pollut. 215, 58–65. doi: 10.1016/j.envpol.2016.04.013, PMID: 27179324

[ref27] Pu Q. Zhao L.-X. Li Y.-T. Su J.-Q. (2020). Manure fertilization increase antibiotic resistance in soils from typical greenhouse vegetable production bases. J. Hazard Mater 391:122267. doi: 10.1016/j.jhazmat.2020.122267, PMID: 32062545

[ref28] Seidlerova Z. Kubasova T. Faldynova M. Crhanova M. Karasova D. Babak V.. (2020). Environmental impact on differential composition of gut microbiota in indoor chickens in commercial production and outdoor, backyard chickens. Microorganisms 8:767. doi: 10.3390/microorganisms8050767, PMID: 32443788 PMC7285315

[ref29] Tahamtani F. M. Kittelsen K. Vasdal G. (2022). Environmental enrichment in commercial flocks of aviary housed laying hens: relationship with plumage condition and fearfulness. Poult. Sci. 101:101754. doi: 10.1016/j.psj.2022.101754, PMID: 35245804 PMC8892153

[ref30] Tamburini S. Shen N. Wu H. C. Clemente J. C. (2016). The microbiome in early life: implications for health outcomes. Nat. Med. 22, 713–722. doi: 10.1038/nm.4142, PMID: 27387886

[ref31] Van Boeckel T. P. Pires J. Silvester R. Zhao C. Song J. Criscuolo N. G.. (2019). Global trends in antimicrobial resistance in animals in low-and middle-income countries. Science 365:eaaw1944. doi: 10.1126/science.aaw1944 31604207

[ref25] Videnska P. Sedlar K. Lukac M. Faldynova M. Gerzova L. Cejkova D.. (2014). Succession and replacement of bacterial populations in the Caecum of egg laying hens over their whole life. PLoS One 9:e115142. doi: 10.1371/journal.pone.0115142, PMID: 25501990 PMC4264878

[ref32] Volf J. Crhanova M. Karasova D. Faldynova M. Kubasova T. Seidlerova Z.. (2021). Eggshell and feed microbiota do not represent major sources of gut anaerobes for chickens in commercial production. Microorganisms 9:1480. doi: 10.3390/microorganisms9071480, PMID: 34361916 PMC8305510

[ref33] Wang Z. Han M. Li E. Liu X. Ning K. (2020). Distribution of antibiotic resistance genes in an agriculturally disturbed lake in China: their links with microbial communities, antibiotics, and water quality. J. Hazard. Mater. 393:122426. doi: 10.1016/j.jhazmat.2020.122426 32143164

[ref34] Wang Y. Hu Y. Cao J. Bi Y. Lv N. Liu F.. (2019). Antibiotic resistance gene reservoir in live poultry markets. J. Infect. 78, 445–453. doi: 10.1016/j.jinf.2019.03.012, PMID: 30935879

[ref35] Wang L. Mike L. Yu Z. (2016). Intestinal microbiota of broiler chickens as affected by litter management regimens. Front. Microbiol. 7:593. doi: 10.3389/fmicb.2016.00593, PMID: 27242676 PMC4870231

[ref36] Xiao S. Mi J. Mei L. Liang J. Feng K. Wu Y.. (2021). Microbial diversity and community variation in the intestines of layer chickens. Animals 11:840. doi: 10.3390/ani11030840, PMID: 33809729 PMC8002243

[ref37] Yang Y. Chen N. Sun L. Zhang Y. Wu Y. Wang Y.. (2021). Short-term cold stress can reduce the abundance of antibiotic resistance genes in the cecum and feces in a pig model. J. Hazard. Mater. 416:125868. doi: 10.1016/j.jhazmat.2021.125868, PMID: 34492815

[ref38] Yang Y. Jiang X. Chai B. Ma L. Li B. Zhang A.. (2016). ARGs-OAP: online analysis pipeline for antibiotic resistance genes detection from metagenomic data using an integrated structured ARG-database. Bioinformatics 32, 2346–2351. doi: 10.1093/bioinformatics/btw136, PMID: 27153579

[ref39] Yin X. Jiang X. Chai B. Li L. Yang Y. Cole J.. (2018). ARGs-OAP v2.0 with an expanded SARG database and hidden Markov models for enhancement characterization and quantification of antibiotic resistance genes in environmental metagenomes. Bioinformatics 34, 2263–2270. doi: 10.1093/bioinformatics/bty053, PMID: 29408954

[ref40] Zhang S. Abbas M. Rehman M. Huang Y. Zhou R. Gong S.. (2020). Dissemination of antibiotic resistance genes (ARGs) via integrons in *Escherichia coli*: a risk to human health. Environ. Pollut. 266:115260. doi: 10.1016/j.envpol.2020.115260, PMID: 32717638

[ref41] Zhao Y. Su J.-Q. An X.-L. Huang F.-Y. Rensing C. Brandt K. K.. (2018). Feed additives shift gut microbiota and enrich antibiotic resistance in swine gut. Sci. Total Environ. 621, 1224–1232. doi: 10.1016/j.scitotenv.2017.10.106, PMID: 29054657

[ref42] Zhou Y. Li Y. Zhang L. Wu Z. Wang H. H. (2020). Antibiotic administration routes and Oral exposure to antibiotic resistant Bacteria as key drivers for gut microbiota disruption and Resistome in poultry. Front. Microbiol. 11:1319. doi: 10.3389/fmicb.2020.01319 32733394 PMC7358366

[ref43] Zhu T. Chen T. Cao Z. Zhong S. Wen X. Mi J.. (2021). Antibiotic resistance genes in layer farms and their correlation with environmental samples. Poult. Sci. 100:101485. doi: 10.1016/j.psj.2021.101485, PMID: 34695626 PMC8554274

