# Supplementary material for: The abundance and diversity of antibiotic resistance genes in layer chicken ceca is associated with farm enviroment
Source: Front Microbiol. 2023 Jun 30;14:1177404. doi: 10.3389/fmicb.2023.1177404 (PMC10348872; doi:10.3389/fmicb.2023.1177404)
Supplement: Supplementary file 1 [file Data_Sheet_1.docx]

Supplementary Material

**The abundance and diversity of antibiotic resistance genes in layer chicken cecal is associated with farm environment**

Shasha Xiao^1^, Jiandui Mi^123^, Yingxin Chen^1^, Kunxian Feng^1^, Liang Mei^1^, Xindi Liao^123^, Yinbao Wu^123^, Yan Wang^123^*

**Correspondence: Yan Wang**Corresponding Author
ywang@scau.edu.cn

# Supplementary Figures and Tables

## Supplementary Figures


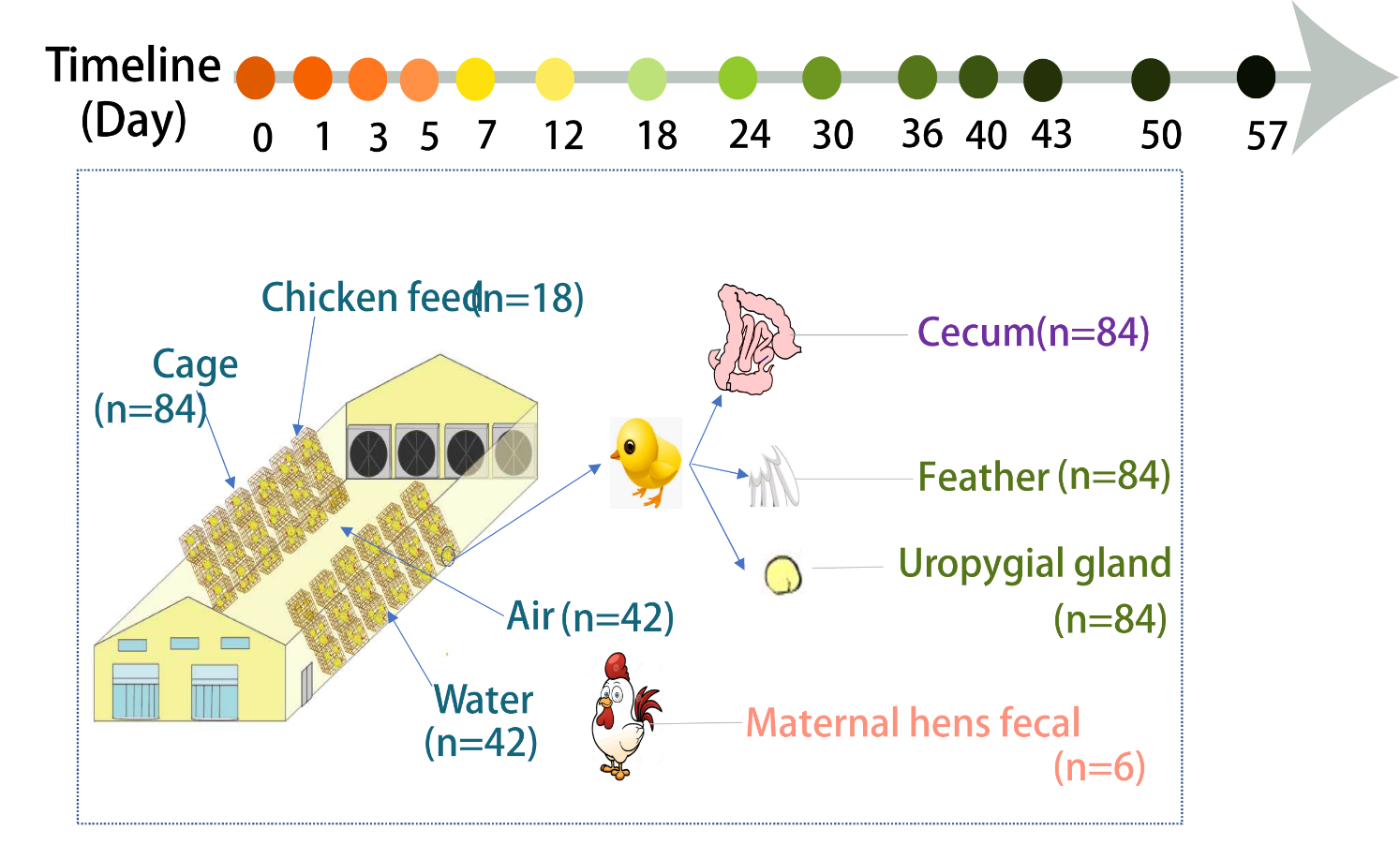


**Supplementary Figure 1**. A longitudinal exploration of microbiomes between both laying chickens, maternal hens fecal and housing environment.


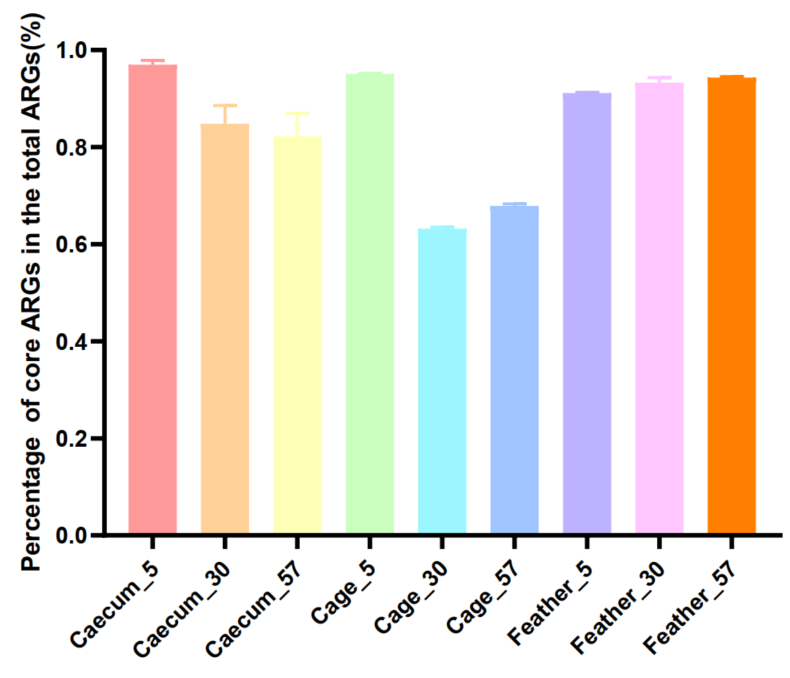


**Supplementary Figure 2.** Percentage of core ARGs in the total ARGs. The height of the bar represents the average value of the core gene set in a group of samples in the overall resistance group, and the error bar represents the standard error within the group.


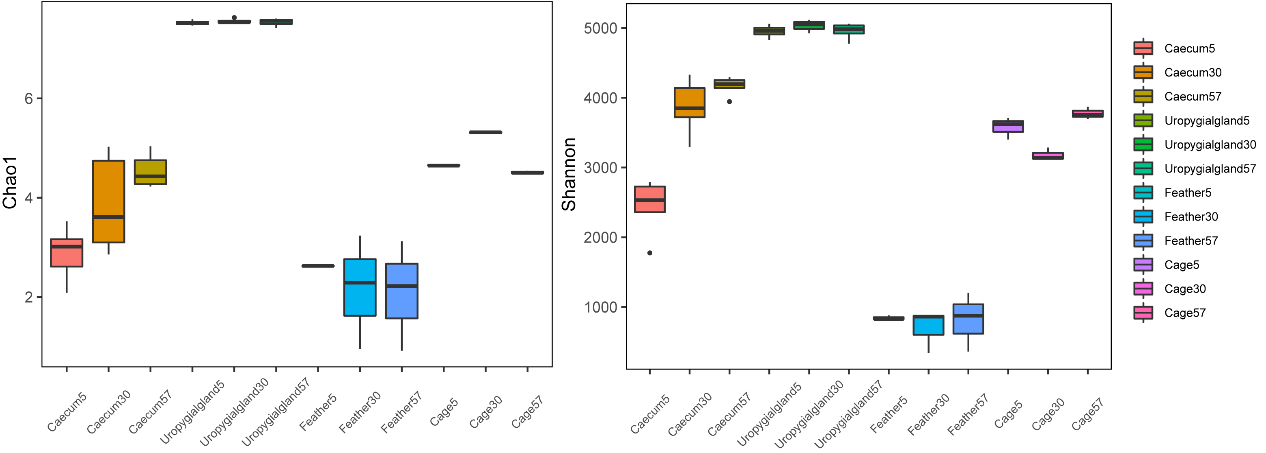


**Supplementary Figure 3.** Longitudinal exploration of microbiome between both Roman powder layer chickens and housing environment. The alpha diversity indices (Chao1, Shannon) with age across layer chickens caecum and environment.


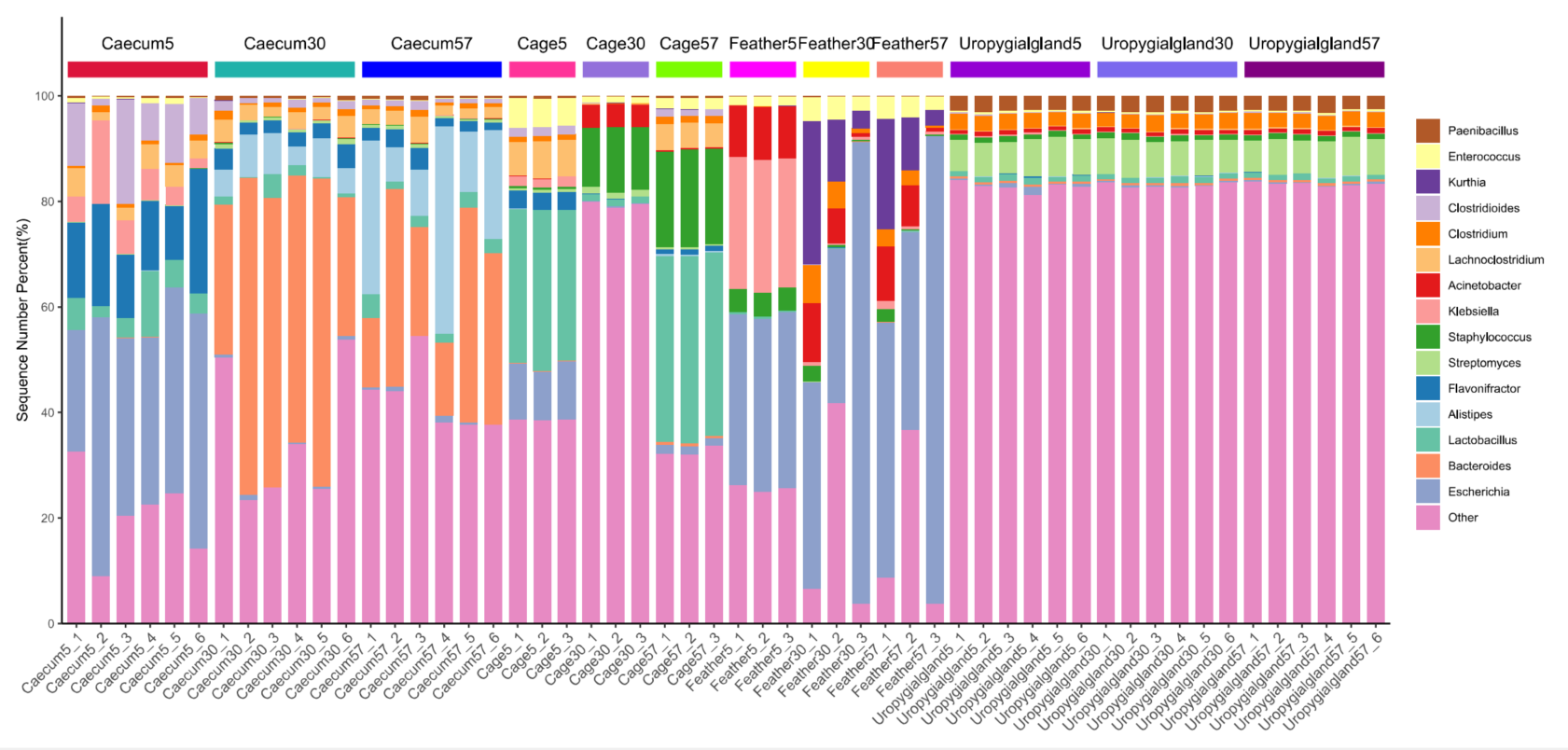


**Supplementary Figure 4.** Relative abundance of the genus in different types sample. Stacked bar chart displaying the change in the average relative abundance of the top10 genus in layer chicken and housing environment.


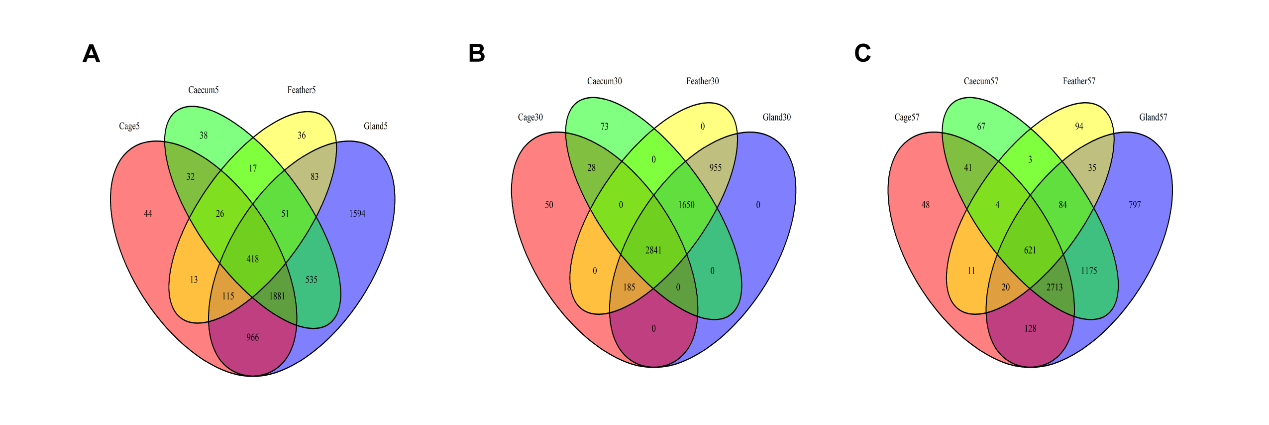


**Supplementary Figure 5.** Shared species among all samples. [Venn diagram](https://www.sciencedirect.com/topics/earth-and-planetary-sciences/venn-diagram) showing the number of shared and unique species among different environments on day5(a), day30(b), day57(c).


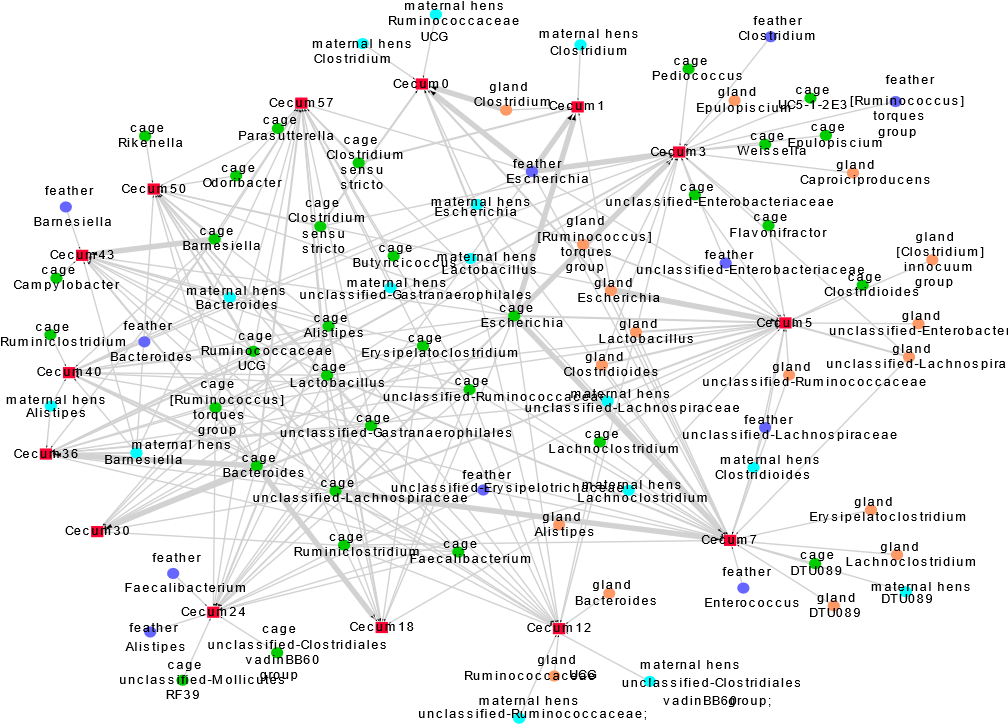


**Supplementary Figure 6.** Source of laying hen caecum microbiota at 14 sampling times. Red square depicts the intestine (different days is displayed in the center of each node). round depict transmitted species, color-coded according to environmental types. Connectingarrows represent the transmission events and the edge thickness is equivalent to the magnitude of the contribution.


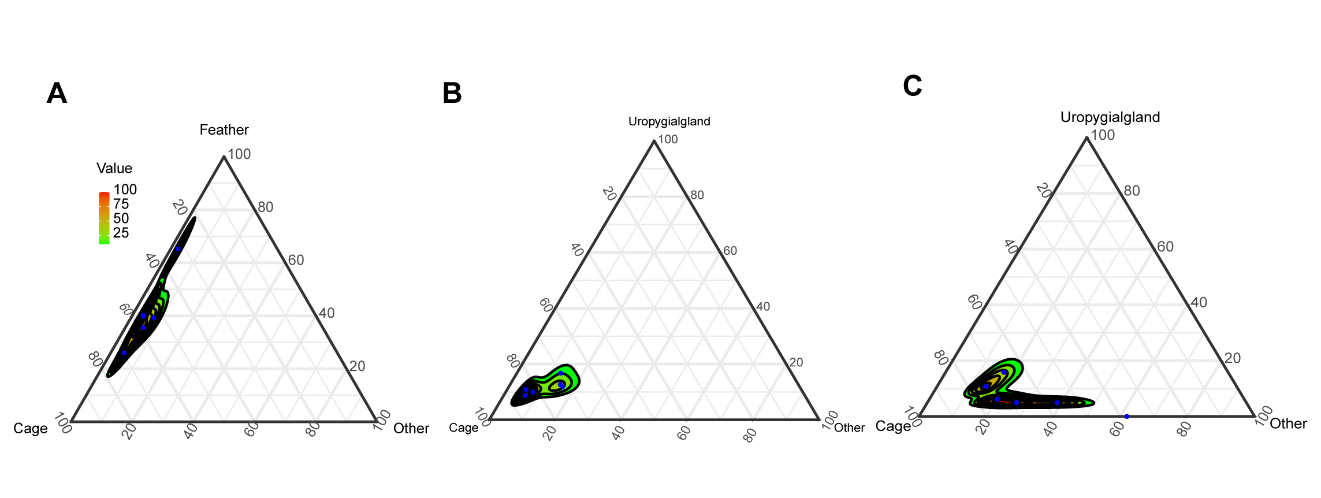


**Supplementary Figure 7.** Source of piglet fecal microbiota at nine sampling times. Three-axis ternary plots indicating the proportion of ASVs within a layer chickens caecum sample (each point) that is predicted to originate from environmental sample (indicated by the triangle vertices). Each blue point represents a layer chickens caecum sample, and its position indicates the predicated relative contribution from the cage, feather or Uropygial gland. The two most important microbial sources for each sampling time were selected as vertices, and the rest sources were labeled as “others”. Points closer to the vertices indicate that a greater proportion of the sample’s ASVs are predicated to originate from the microbiota of the indicated environmental sample.

## Supplementary Table

**Supplementary Table 1** Top 10 most abundant ARG subtypes

| ARGs subtype | Sample | Day |
| --- | --- | --- |
| aminoglycoside__aadE | caecum | day5 |
| aminoglycoside__aph(3'')-I |  |  |
| aminoglycoside__aph(6)-I |  |  |
| bacitracin__bacA |  |  |
| multidrug__acrA |  |  |
| multidrug__acrB |  |  |
| multidrug__emrB |  |  |
| multidrug__mdtL |  |  |
| tetracycline__tetA |  |  |
| tetracycline__tetW |  |  |
| aminoglycoside__aadE | cage |  |
| aminoglycoside__aph(3''')-III |  |  |
| bacitracin__bacA |  |  |
| macrolide-lincosamide-streptogramin__ermB |  |  |
| tetracycline__tet40 |  |  |
| tetracycline__tetA |  |  |
| tetracycline__tetM |  |  |
| tetracycline__tetO |  |  |
| tetracycline__tetW |  |  |
| vancomycin__vanR |  |  |
| aminoglycoside__aph(3'')-I | feather |  |
| aminoglycoside__aph(6)-I |  |  |
| bacitracin__bacA |  |  |
| multidrug__acrA |  |  |
| multidrug__acrB |  |  |
| multidrug__emrA |  |  |
| multidrug__emrB |  |  |
| multidrug__TolC |  |  |
| sulfonamide__sul1 |  |  |
| tetracycline__tetA |  |  |
| aminoglycoside__aadE | caecum | day30 |
| aminoglycoside__aph(3''')-III |  |  |
| macrolide-lincosamide-streptogramin__ermB |  |  |
| macrolide-lincosamide-streptogramin__ermF |  |  |
| macrolide-lincosamide-streptogramin__ermG |  |  |
| tetracycline__tet32 |  |  |
| tetracycline__tet40 |  |  |
| tetracycline__tet44 |  |  |
| tetracycline__tetQ |  |  |
| tetracycline__tetW |  |  |
| aminoglycoside__aadA | cage |  |
| aminoglycoside__ant(9)-I |  |  |
| aminoglycoside__aph(3'')-I |  |  |
| aminoglycoside__aph(6)-I |  |  |
| chloramphenicol__floR |  |  |
| macrolide-lincosamide-streptogramin__lnuA |  |  |
| multidrug__qacEdelta1 |  |  |
| multidrug__qacG |  |  |
| sulfonamide__sul1 |  |  |
| tetracycline__tetZ |  |  |
| aminoglycoside__aph(3'')-I | feather |  |
| bacitracin__bacA |  |  |
| multidrug__acrB |  |  |
| multidrug__emrA |  |  |
| multidrug__emrB |  |  |
| multidrug__mdtA |  |  |
| multidrug__mdtE |  |  |
| multidrug__mdtM |  |  |
| multidrug__mdtP |  |  |
| tetracycline__tetA |  |  |
| aminoglycoside__aadE | caecum | day57 |
| aminoglycoside__aph(3''')-III |  |  |
| macrolide-lincosamide-streptogramin__ermB |  |  |
| macrolide-lincosamide-streptogramin__ermF |  |  |
| macrolide-lincosamide-streptogramin__vatB |  |  |
| tetracycline__tet32 |  |  |
| tetracycline__tet44 |  |  |
| tetracycline__tetM |  |  |
| tetracycline__tetQ |  |  |
| tetracycline__tetW |  |  |
| aminoglycoside__aadE | cage |  |
| aminoglycoside__ant(9)-I |  |  |
| aminoglycoside__aph(3''')-III |  |  |
| macrolide-lincosamide-streptogramin__ermB |  |  |
| macrolide-lincosamide-streptogramin__lnuA |  |  |
| multidrug__qacG |  |  |
| tetracycline__tet44 |  |  |
| tetracycline__tetL |  |  |
| tetracycline__tetM |  |  |
| tetracycline__tetW |  |  |
| aminoglycoside__aph(3'')-I | feather |  |
| bacitracin__bacA |  |  |
| multidrug__acrB |  |  |
| multidrug__emrA |  |  |
| multidrug__emrB |  |  |
| multidrug__mdtA |  |  |
| multidrug__mdtN |  |  |
| multidrug__mdtO |  |  |
| multidrug__mdtP |  |  |
| tetracycline__tetA |  |  |

**Supplementary Table 2** ARG subtypes shared by layer chickens caecum feather and cage

| ARG types | ARG subtypes |
| --- | --- |
| aminoglycoside | aac(6')-I |
| aminoglycoside | aad(6) |
| aminoglycoside | aadA |
| aminoglycoside | aadE |
| aminoglycoside | aph(3')-I |
| aminoglycoside | aph(3'')-I |
| aminoglycoside | aph(3''')-III |
| aminoglycoside | aph(6)-I |
| aminoglycoside | bifunctional aminoglycoside N-acetyltransferase and aminoglycoside phosphotransferase |
| bacitracin | bacA |
| bacitracin | bcrA |
| beta-lactam | ampC |
| beta-lactam | class A beta-lactamase |
| beta-lactam | class C beta-lactamase |
| beta-lactam | CMY-2 |
| beta-lactam | CMY-41 |
| beta-lactam | CMY-99 |
| beta-lactam | CTX-M |
| beta-lactam | metallo-beta-lactamase |
| beta-lactam | OXA-10 |
| beta-lactam | OXA-13 |
| beta-lactam | PBP-1A |
| beta-lactam | TEM-1 |
| beta-lactam | TEM-117 |
| beta-lactam | TEM-118 |
| beta-lactam | TEM-178 |
| beta-lactam | TEM-187 |
| beta-lactam | TEM-195 |
| beta-lactam | TEM-205 |
| beta-lactam | TEM-209 |
| beta-lactam | TEM-75 |
| beta-lactam | TEM-91 |
| bleomycin | bleomycin resistance protein |
| chloramphenicol | cat-chloramphenicol acetyltransferase |
| chloramphenicol | catD |
| chloramphenicol | chloramphenicol and florfenicol exporter |
| chloramphenicol | chloramphenicol and florfenicol resistance gene |
| chloramphenicol | chloramphenicol exporter |
| chloramphenicol | cmlA |
| chloramphenicol | floR |
| fosfomycin | fosX |
| fosmidomycin | rosA |
| fosmidomycin | rosB |
| kasugamycin | kasugamycin resistance protein ksgA |
| macrolide-lincosamide-streptogramin | ermB |
| macrolide-lincosamide-streptogramin | ermG |
| macrolide-lincosamide-streptogramin | ermT |
| macrolide-lincosamide-streptogramin | lsa |
| macrolide-lincosamide-streptogramin | macA |
| macrolide-lincosamide-streptogramin | macB |
| macrolide-lincosamide-streptogramin | mefA |
| macrolide-lincosamide-streptogramin | mphA |
| macrolide-lincosamide-streptogramin | vatB |
| multidrug | acrA |
| multidrug | acrB |
| multidrug | acrF |
| multidrug | adeJ |
| multidrug | bicyclomycin-multidrug efflux protein bcr |
| multidrug | emrA |
| multidrug | emrB |
| multidrug | EmrB-QacA family major facilitator transporter |
| multidrug | emrD |
| multidrug | emrE |
| multidrug | emrK |
| multidrug | mdfA |
| multidrug | mdtA |
| multidrug | mdtB |
| multidrug | mdtC |
| multidrug | mdtD |
| multidrug | mdtE |
| multidrug | mdtF |
| multidrug | mdtG |
| multidrug | mdtH |
| multidrug | mdtK |
| multidrug | mdtL |
| multidrug | mdtM |
| multidrug | mdtN |
| multidrug | mdtO |
| multidrug | mdtP |
| multidrug | mexB |
| multidrug | mexD |
| multidrug | mexE |
| multidrug | mexF |
| multidrug | multidrug-ABC-transporter |
| multidrug | multidrug-transporter |
| multidrug | qacEdelta1 |
| multidrug | TolC |
| polymyxin | arnA |
| quinolone | qnrS |
| rifamycin | ADP-ribosylating transferase arr |
| sulfonamide | sul1 |
| sulfonamide | sul2 |
| tetracycline | tet32 |
| tetracycline | tet34 |
| tetracycline | tet35 |
| tetracycline | tet40 |
| tetracycline | tet44 |
| tetracycline | tetA |
| tetracycline | tetC |
| tetracycline | tetL |
| tetracycline | tetM |
| tetracycline | tetO |
| tetracycline | tetP |
| tetracycline | tetracycline resistance protein |
| tetracycline | tetW |
| trimethoprim | dfrA14 |
| trimethoprim | dfrA16 |
| trimethoprim | dfrA17 |
| unclassified | antibiotic resistance rRNA adenine methyltransferase |
| unclassified | bacterial regulatory protein LuxR |
| unclassified | cAMP-regulatory protein |
| unclassified | cob(I)alamin adenolsyltransferase |
| unclassified | DNA-binding transcriptional regulator gadX |
| unclassified | DNA-binding protein H-NS |
| unclassified | sdiA |
| unclassified | transcriptional regulatory protein CpxR cpxR |
| unclassified | truncated putative response regulator ArlR |
| vancomycin | vanC |
| vancomycin | vanD |
| vancomycin | vanG |
| vancomycin | vanR |
| vancomycin | vanS |
| vancomycin | vanY |

**Supplementary Table 3** ARGs contributed more than 0.1%

| aminoglycoside | aac(3)-II |
| --- | --- |
| aminoglycoside | aac(3)-IV |
| aminoglycoside | aac(6')-I |
| aminoglycoside | aad(9) |
| aminoglycoside | aadD |
| aminoglycoside | aadK |
| aminoglycoside | ant(2'')-I |
| aminoglycoside | ant(3'')-Ih-aac(6')-IId |
| aminoglycoside | aph(2'')-II |
| aminoglycoside | aph(2'')-III |
| aminoglycoside | aph(2'')-IV |
| aminoglycoside | aph(3')-I |
| aminoglycoside | aph(3')-VII |
| aminoglycoside | aph(4)-I |
| aminoglycoside | rmtD |
| beta-lactam | ampC |
| beta-lactam | ccrA |
| beta-lactam | CFE-1 |
| beta-lactam | CfxA2 |
| beta-lactam | CMY-100 |
| beta-lactam | CMY-102 |
| beta-lactam | CMY-103 |
| beta-lactam | CMY-111 |
| beta-lactam | CMY-13 |
| beta-lactam | CMY-16 |
| beta-lactam | CMY-2 |
| beta-lactam | CMY-21 |
| beta-lactam | CMY-25 |
| beta-lactam | CMY-26 |
| beta-lactam | CMY-29 |
| beta-lactam | CMY-32 |
| beta-lactam | CMY-37 |
| beta-lactam | CMY-39 |
| beta-lactam | CMY-4 |
| beta-lactam | CMY-40 |
| beta-lactam | CMY-41 |
| beta-lactam | CMY-43 |
| beta-lactam | CMY-51 |
| beta-lactam | CMY-55 |
| beta-lactam | CMY-6 |
| beta-lactam | CMY-60 |
| beta-lactam | CMY-61 |
| beta-lactam | CMY-62 |
| beta-lactam | CMY-64 |
| beta-lactam | CMY-73 |
| beta-lactam | CMY-74 |
| beta-lactam | CMY-78 |
| beta-lactam | CMY-99 |
| beta-lactam | CTX-M |
| beta-lactam | CTX-M-1 |
| beta-lactam | CTX-M-101 |
| beta-lactam | CTX-M-110 |
| beta-lactam | CTX-M-114 |
| beta-lactam | CTX-M-12 |
| beta-lactam | CTX-M-132 |
| beta-lactam | CTX-M-14 |
| beta-lactam | CTX-M-142 |
| beta-lactam | CTX-M-147 |
| beta-lactam | CTX-M-19 |
| beta-lactam | CTX-M-22 |
| beta-lactam | CTX-M-28 |
| beta-lactam | CTX-M-29 |
| beta-lactam | CTX-M-32 |
| beta-lactam | CTX-M-42 |
| beta-lactam | CTX-M-51 |
| beta-lactam | CTX-M-52 |
| beta-lactam | CTX-M-53 |
| beta-lactam | CTX-M-54 |
| beta-lactam | CTX-M-55 |
| beta-lactam | CTX-M-58 |
| beta-lactam | CTX-M-60 |
| beta-lactam | CTX-M-61 |
| beta-lactam | CTX-M-62 |
| beta-lactam | CTX-M-69 |
| beta-lactam | CTX-M-79 |
| beta-lactam | CTX-M-82 |
| beta-lactam | CTX-M-99 |
| beta-lactam | LEN-19 |
| beta-lactam | OKP-B |
| beta-lactam | OXA-1 |
| beta-lactam | OXA-10 |
| beta-lactam | OXA-13 |
| beta-lactam | OXA-142 |
| beta-lactam | OXA-147 |
| beta-lactam | OXA-17 |
| beta-lactam | OXA-19 |
| beta-lactam | OXA-251 |
| beta-lactam | OXA-28 |
| beta-lactam | OXA-35 |
| beta-lactam | OXA-4 |
| beta-lactam | OXA-9 |
| beta-lactam | PBP-1A |
| beta-lactam | PBP-1B |
| beta-lactam | SHV-1 |
| beta-lactam | SHV-112 |
| beta-lactam | SHV-12 |
| beta-lactam | SHV-121 |
| beta-lactam | SHV-128 |
| beta-lactam | SHV-142 |
| beta-lactam | SHV-147 |
| beta-lactam | SHV-152 |
| beta-lactam | SHV-154 |
| beta-lactam | SHV-162 |
| beta-lactam | SHV-167 |
| beta-lactam | SHV-28 |
| beta-lactam | SHV-37 |
| beta-lactam | SHV-4 |
| beta-lactam | SHV-51 |
| beta-lactam | SHV-53 |
| beta-lactam | SHV-6 |
| beta-lactam | SHV-71 |
| beta-lactam | SHV-93 |
| beta-lactam | TEM-1 |
| beta-lactam | TEM-101 |
| beta-lactam | TEM-106 |
| beta-lactam | TEM-108 |
| beta-lactam | TEM-117 |
| beta-lactam | TEM-118 |
| beta-lactam | TEM-123 |
| beta-lactam | TEM-127 |
| beta-lactam | TEM-138 |
| beta-lactam | TEM-139 |
| beta-lactam | TEM-144 |
| beta-lactam | TEM-146 |
| beta-lactam | TEM-153 |
| beta-lactam | TEM-154 |
| beta-lactam | TEM-156 |
| beta-lactam | TEM-157 |
| beta-lactam | TEM-162 |
| beta-lactam | TEM-177 |
| beta-lactam | TEM-178 |
| beta-lactam | TEM-183 |
| beta-lactam | TEM-184 |
| beta-lactam | TEM-186 |
| beta-lactam | TEM-187 |
| beta-lactam | TEM-194 |
| beta-lactam | TEM-195 |
| beta-lactam | TEM-205 |
| beta-lactam | TEM-209 |
| beta-lactam | TEM-21 |
| beta-lactam | TEM-213 |
| beta-lactam | TEM-29 |
| beta-lactam | TEM-43 |
| beta-lactam | TEM-52 |
| beta-lactam | TEM-53 |
| beta-lactam | TEM-6 |
| beta-lactam | TEM-63 |
| beta-lactam | TEM-75 |
| beta-lactam | TEM-78 |
| beta-lactam | TEM-83 |
| beta-lactam | TEM-89 |
| beta-lactam | TEM-91 |
| beta-lactam | TEM-95 |
| chloramphenicol | catB |
| chloramphenicol | catS |
| chloramphenicol | cmlA |
| fosfomycin | fosA |
| fosfomycin | fosX |
| fosmidomycin | rosB |
| MLS | ermC |
| MLS | ermF |
| MLS | lmrB |
| MLS | lnuB |
| MLS | mphA |
| MLS | msrC |
| MLS | vatC |
| MLS | vatD |
| MLS | vatE |
| MLS | vatG |
| MLS | vgaD |
| multidrug | acrA |
| multidrug | acrF |
| multidrug | adeB |
| multidrug | adeC |
| multidrug | adeJ |
| multidrug | amrB |
| multidrug | ceoB |
| multidrug | cmeB |
| multidrug | emrK |
| multidrug | mdfA |
| multidrug | mdtA |
| multidrug | mdtB |
| multidrug | mdtC |
| multidrug | mdtD |
| multidrug | mdtE |
| multidrug | mdtG |
| multidrug | mdtH |
| multidrug | mdtL |
| multidrug | mdtP |
| multidrug | mexA |
| multidrug | mexB |
| multidrug | mexD |
| multidrug | mexE |
| multidrug | mexF |
| multidrug | mexI |
| multidrug | mexW |
| multidrug | mexY |
| multidrug | mtrE |
| multidrug | oprC |
| multidrug | oprM |
| multidrug | qacB |
| multidrug | sdeY |
| multidrug | smeB |
| multidrug | smeE |
| multidrug | smeF |
| quinolone | norB |
| quinolone | qnrS |
| tetracycline | otrA |
| tetracycline | tet34 |
| tetracycline | tet35 |
| tetracycline | tet36 |
| tetracycline | tetB |
| tetracycline | tetC |
| tetracycline | tetD |
| tetracycline | tetK |
| tetracycline | tetP |
| tetracycline | tetQ |
| tetracycline | tetS |
| tetracycline | tetX |
| tetracycline | tetX3 |
| tetracycline | tetX4 |
| tetracycline | tetX5 |
| tetracycline | tetX6 |
| trimethoprim | dfrA12 |
| trimethoprim | dfrA14 |
| trimethoprim | dfrA16 |
| trimethoprim | dfrA17 |
| trimethoprim | dfrA25 |
| trimethoprim | dfrA5 |
| vancomycin | vanA |
| vancomycin | vanB |
| vancomycin | vanC |
| vancomycin | vanD |
| vancomycin | vanH |
| vancomycin | vanN |
| vancomycin | vanT |
| vancomycin | vanU |
| vancomycin | vanW |
| vancomycin | vanX |
| vancomycin | vanY |
